# Red edge effect of chalcone derivatives and their application in Bio-sensing†

**DOI:** 10.1039/d4ra06978a

**Published:** 2025-04-28

**Authors:** Amrita Saha, Monaj Karar, Sudip Choudhury

**Affiliations:** a MLR Institute of Technology Hyderabad Telangana 500043 India drmonajkarar@mlrinstitutions.ac.in; b Department of Chemistry, Assam University Silchar-788 011 India dramrita@mlrinstitutions.ac.in

## Abstract

A series of chalcone-based compounds with varied functional groups were designed and synthesized through green chemistry. Polarity-tuned solvatochromic photophysical studies were thoroughly performed using steady-state absorption and emission spectroscopic techniques. These donor–acceptor structured chalcones were capable of exhibiting excitation dependent fluorescence (EDF), which is widely known as the red edge effect, with a large Stokes shift above 140 nm, making them capable of accessing the yellow to blue region of the spectrum. Furthermore, the ease of tuning the fluorescence property was utilized in the field of biosensors to obtain tunable brilliant blue to green colors for the first time. Computational studies indicated the presence of relatively compact states, which might be one of the key factors responsible for the red edge effect.

## Abstract 2

Chalcones capable of exhibiting excitation dependent fluorescence (EDF) and its EDF was utilized in the field of biosensors to obtain tunable brilliant blue to green color.

## Graphical abstract

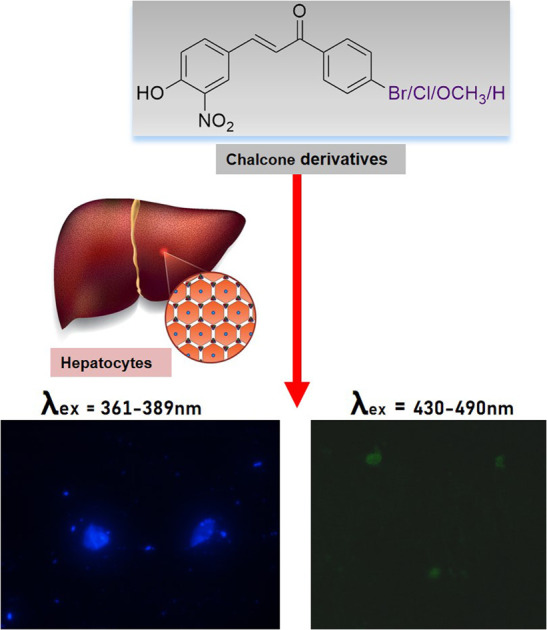

## 1. Introduction

Excitation dependent fluorescence (EDF), popularly known as the Red-edge effect, is an uncommon fluorescence exhibited by some polar molecules, wherein the fluorescence bands shift toward longer wavelengths with an increase in the excitation wavelength.^1,2^ This effect has received extensive attention owing to their unique molecular relaxation dynamics and applications in both biological and material sciences. Recently, a very high red-edge effect (up to 200 nm) has been reported for functionalized graphene oxide in a polar solvent; the effect was not observed in nonpolar solvents.^3^

Tunable photoluminescence properties, including the Red Edge effect, have been studied since the 1970s and continues to be explored today,^1,4,5^ which includes numerous molecules-larger systems such as ethyl 5-(4-aminophenyl)-3-amino-2,4-dicyanobenzoate,^6^ phenanthrene,^7^ and *meso*-tetraphenyl porphyrin^8^ have been extensively studied for this effect. If the tunable photoluminescence of small organic fluorophores is reciprocated in bio-sensing application, it will be beneficial in the fields of medical diagnostics, anti-bioterrorism, and food safety.^9^ However, research works^10^ are scarce where attempts have been made to apply the Red Edge effect of small organic molecules in bio-sensing applications.

Researches on 1, 2-dihydropyrrolo [3,4-*b*]indolizin-3-one derivatives demonstrated an emission wavelength of 420–613 nm where indolizine core-based probes stained A375 cells only at a specific wavelength.^11^ The majority of such fluorophores are solvatochromic but exhibit a short and strict excitation-emission window. Combined with complex synthetic procedures, such probes are unsuitable if any of the background material's fluorescence is at that excitation wavelength. To solve these issues and to incorporate the wide range of sound features associated with EDF into small organic fluorophore molecules, development of new biosensor molecules would be of great interest.

**Fig. 1:**
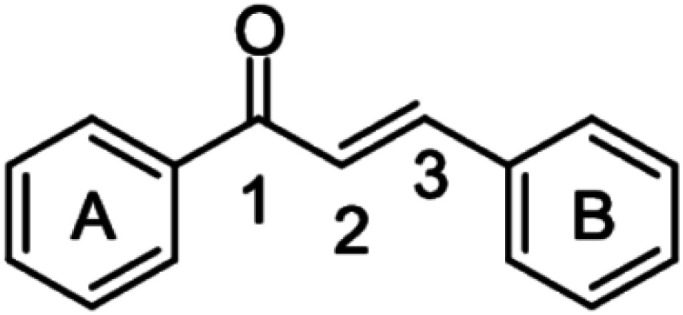
Fundamental structure of chalcone derivatives.

**Chart 1:**
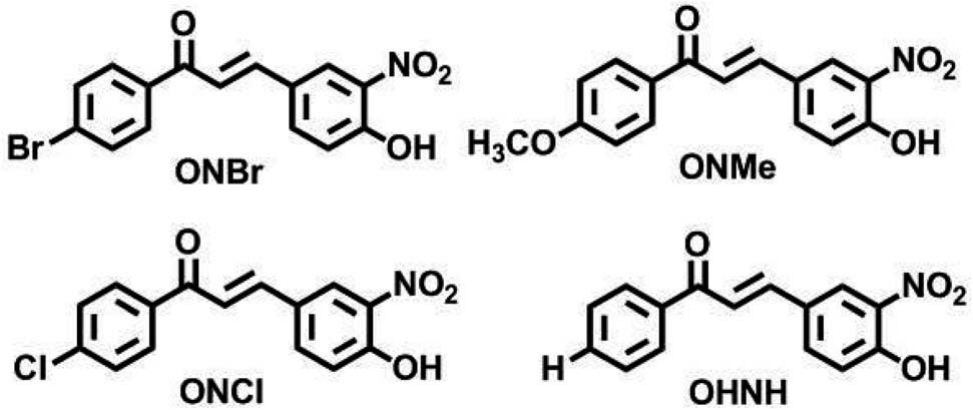
Structures of the synthesized ONBr, ONMe, ONCl and OHNH compounds.

To exhibit the red edge effect, there should be a difference in the dipole moment between the ground state and the electronically excited state of the fluorophore. In addition, good water solubility is a primary requirement for bioprobes.^12^ To satisfy the aforementioned criteria, a series of fluorophores were synthesized with donor–Acceptor (D–A) chalcones so that internal charge transfer (ICT) would occur. To tune the fluorescence,^13,14^ the positions of the –NO_2_ and –OH groups were fixed on the B ring of the chalcone moiety (Fig. 1), and the substituents on the A ring (the phenyl ring to which the carbonyl is attached) were varied. The -Br, -Cl, -H, and -OMe substituents were chosen to determine how the electron-donating and electron-withdrawing groups can cause changes in the EDF property as well as in the sensing property. As mentioned, Ring B was fixed with –NO_2_ and –OH groups, as in the presence of the –OH group, hydrogen bonding can occur with the solvent. At first, –H group was introduced in Ring A, or in other words, Ring A was left un-substituted to determine the sole effect of –NO_2_ and –OH in Ring B. In the next scenario, Ring A was substituted with the –Cl and –Br groups as these two groups have both +R and −I effects, and this could craft the electronic push–pull. Lastly, the –OCH_3_ group was substituted in Ring A, which has an intense +R effect but no −I effect. The structures of the synthesized compounds are presented in Chart 1.

The control on emission frequency connects these compounds as imperative and functional members in material applications. As the ‘input’ and ‘output’ frequencies/shifts can be somewhat ‘chosen’, they are employed in tailor-made high-contrast fluorophores transducing agents in bio- and chemical-sensing, biological imaging, NLO materials, *etc.*

This study reports the detailed fluorescence properties, solvatochromism (in a polar and nonpolar solvent), and sensing ability of some chalcone derivatives using a cheap, green, one step synthetic method. We preferred chalcone derivatives because of their numerous applications in biological and material science.^15–20^ In addition, computation studies were conducted on this set of compounds to understand the observed phenomenon.

## 2. Synthetic procedure and experimental details

### 2.1 Synthesis

**Scheme 1:**
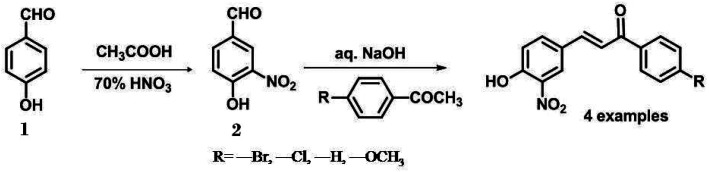
Scheme for the synthesis of 1-(4-substituted)-3-(4-hydroxy-3-nitrophenyl) prop-2-en-1-one.

4-hydroxy-3 nitro benzaldehyde (2) was synthesized by the nitration of 4-hydroxybenzaldehyde (1) by the method mentioned by P. Ionita.^21^ 1-(4-substituted)-3-(4-hydroxy-3-nitrophenyl) prop-2-en-1-one was synthesized using Claisen Schmidt condensation (Scheme 1). In a water bath, a mixture of acetophenone derivative (3.6 mmol) and 4-hydroxy-3-nitrbenzaldehyde (3.6 mmol) was warmed to 30–40 °C in NaOH (40% w/v ethanol and water). After being agitated for 1 to 4 hours, the reaction mixture was left overnight. Thin-layer chromatography was used to track the reaction progress using chloroform: methanol (9 : 1) as the developing solvent. The obtained crimson solution was cautiously acidified by adding strong HCl and decanted into crushed ice. The solid was washed with water until the pH reached neutral. Chalcones were separated as yellow solids, rinsed with water and collected *via* filtration.^22^ Chloroform was used as the eluent in silica gel column chromatography (60–120 mesh) to further purify the synthesized compounds. The characterization NMR spectra of OHNH, ONBr, ONCl, and ONMe are available in the ESI.†

#### 2.1.1 (E)-1-(4-bromophenyl)-3-(4-hydroxy-3-nitrophenyl)prop-2-en-1-one (ONBr)

ONBr was synthesized using the method described in Section 2.1 with 4-bromoacetophenone (199 mg) and 4-hydroxy-3-nitrbenzaldehyde (169 mg). Chloroform: methanol (9 : 1) was used as the developing solvent in TLC. The compound was purified using a silica gel column (60–120 mesh) chromatography with chloroform as the eluent. A yellow, shiny solid was obtained. Yield: 245 mg (70%). ^1^H NMR (400 MHz, CDCl_3_) *δ* 7.23 (d, *J* = 8 Hz, 1H), 7.47 (d, *J* = 15.6 Hz, 1H), 7.78 (d, *J* = 15.6 Hz, 1H), 7.89–7.86 (dd, *J* = 2 Hz and *J* = 2 Hz, 1H), 7.87–7.91 (m, 4H), 8.39 (s, 1H), 10.76 (s, 1H).^22^^13^C NMR (CDCl_3_) *δ* 188.6, 156.4, 142.1, 136.6, 136.5, 132.1, 130.0, 127.5, 121.1, 121.9, 120.9.

#### 2.1.2 (E)-1-(4-chlorophenyl)-3-(4-hydroxy-3-nitrophenyl)prop-2-en-1-one (ONCl)

ONCl was synthesized following the method described in Section 2.1 with 4-chloroacetophenone (155 mg) and 4-hydroxy-3-nitrbenzaldehyde (169 mg). Chloroform: methanol (9 : 1) was used as a developing solvent in TLC. Silica gel column (60–120 mesh) chromatography with chloroform as an eluent was used to purify the compounds, producing a yellow, shiny solid. Yield: 199 mg (65%). ^1^H NMR (400 MHz, CDCl_3_) *δ* 10.76 (s, 1H), 8.39 (s, 1H), 7.99–7.95 (m, 4H), 7.89–7.87 (dd, *J* = 2 Hz and *J* = 2 Hz, 1H), 7.78 (d, *J* = 15.6 Hz, 1H), 7.47 (d, *J* = 15.6 Hz, 1H), 7.23 (d, *J* = 8 Hz, 1H).^22^^13^C (CDCl_3_) *δ* 188.4, 188.3, 156.3, 142.0, 136.6, 129.9, 129.8, 129.0, 125.1, 121.9, 120.9.

#### 2.1.3 (E)-3-(4-hydroxy-3-nitrophenyl)-1-(4-methoxyphenyl)prop-2-en-1-one (ONMe)

ONMe was synthesized following the method described in Section 2.1 with 4-methoxyacetophenone (150 mg) and 4-hydroxy-3-nitrbenzaldehyde (169 mg). Chloroform: methanol (9 : 1) was used as the developing solvent in TLC. Silica gel column (60–120 mesh) chromatography with chloroform as an eluent was used to purify the compounds. Occur yellow solid, Yield: 210 mg (70%); ^1^H NMR (400 MHz, CDCl_3_) *δ* 10.74 (s, 1H), 8.37 (s, 1H), 7.89–7.86 (dd, *J* = 2 Hz and *J* = 2 Hz 1H), 7.75 (d, *J* = 15.6 Hz, 1H), 7.47 (d, *J* = 15.6 Hz, 1H), 7.22 (d, *J* = 8 Hz, 1H), 7.01–6.98 (m, 2H), 3.09 (s, 3H).^22^^13^C (CDCl_3_) *δ* 188.5, 187.9, 156.0, 140.7, 136.6, 130.9, 130.6, 127.9, 122.4, 120.8, 113.9, 55.5.

#### 2.1.4 (E)-3-(4-hydroxy-3-nitrophenyl)-1-phenylprop-2-en-1-one (OHNH)

OHNH was synthesized following the method described in Section 2.1 with acetophenone (120 mg) and 4-hydroxy-3-nitrbenzaldehyde (169 mg). Chloroform: methanol (9 : 1) was used as the developing solvent in TLC. Silica gel column (60–120 mesh) chromatography with chloroform as an eluent was used to purify the compound, yellow shiny solid. Yield: 203 mg (75%); ^1^H NMR (400 MHz, CDCl_3_) *δ* 9.96 (s, 1H), 8.39 (s, 1H), 8.17–8.14 (dd, *J* = 2 Hz and *J* = 2 Hz 1H), 7.78 (d, *J* = 16 Hz, 1H), 7.61 (d, *J* = 8 Hz, 2H), 7.56–7.51 (m, 1H), 7.33 (d, *J* = 8 Hz, 1H), 7.28 (d, *J* = 16 Hz, 1H).^22^

### 2.2 Absorption and emission spectra

A Shimadzu UV-1601PC absorption spectrophotometer was used to record steady-state absorption spectra, a PerkinElmer LS 45 spectrofluorometer was used to record fluorescence spectra, and all of the observed spectra were adjusted for the instrument response function. To measure the absorption and emission spectra, a quartz cuvette with a thickness of 10 mm was used.

### 2.3 Computational details

All calculations were performed using the GAUSSIAN 03 package in the BRAF supercomputing environment. The structures of OHNH, ONMe, ONCl and ONBr were fully optimized using Becke's three-parameter hybrid exchange function,^23^ coupled with the Lee–Yang–Parr nonlocal correlation functional (B3LYP)^24^ DFT using 6–31++g (d, p).

### 2.4 Quantum yield measurements

The relative fluorescence quantum yield was measured using Coumarin 1 as a reference with a recognized quantum yield (*Q*) value of 0.73 in ethanol solvent. The area of the emission spectrum was integrated using Origin 8.5 software, and the relative quantum yield was calculated using the well-known equation:^25^1
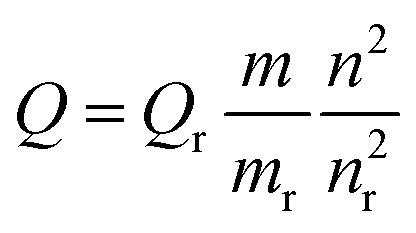
where *Q* and *Q*_r_ represent the fluorescence quantum yields of the sample and reference, respectively; *m* and *m*_r_ are slopes of integrated emission intensity *vs.* absorbance plots for the sample and reference, respectively (at 380 nm excitation wavelength). *n* and *n*_r_ are the refractive indexes of CHCl_3_ and ethanol, respectively.^25^

## 3. Results and discussion

### 3.1 Absorption and emission spectroscopic studies

**Fig. 2:**
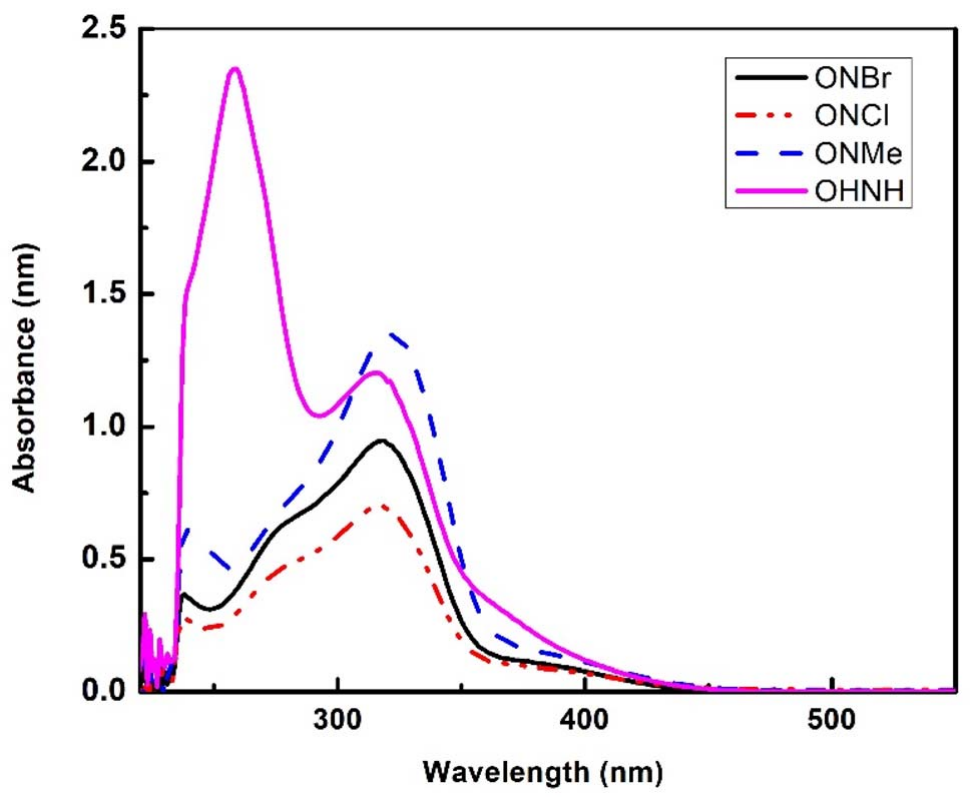
Absorption spectra of OHNH, ONMe, ONCl, and ONBr in chloroform.

The absorption spectra were collected in different solvents with diverse polarity index ranging from 0.1 (hexane), 4.1 (chloroform), 5.1 (acetone) to 5.2 (ethanol), and at ambient temperature with a concentration of 0.5 × 10^−3^ (M). The absorption maxima of all the compounds in various solvents are presented in Table 1, and the absorption spectra in chloroform are shown in Fig. 2 (all other absorption Spectra can be found in ESI Fig. S2.1–S2.4†).

**Table 1:** Photophysical data for OHNH, ONMe, ONCl, and ONBr

Compound	Absorbance maxima (nm)							
	Solvent							
	Hexane		Chloroform		Acetone		Ethanol	
	Π → π*	n–π*	Π → π*	n–π*	Π → π*	N–π*	Π → π*	n–π*
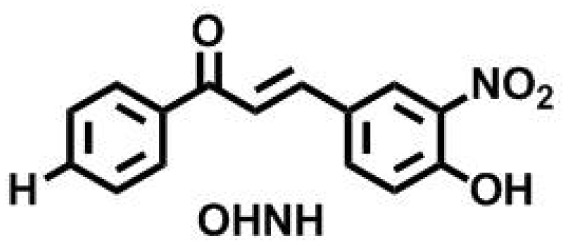	306	360	257	376	329	384	255	325
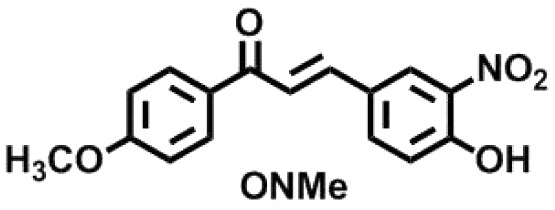	313	376	321	393	330	393	272	325
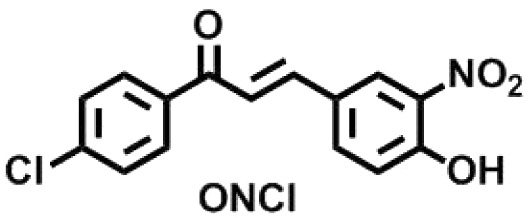	310	375	316	386	328		270	324
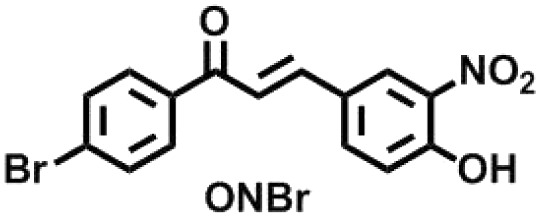	310	378	317	389	330	386	274	322

Two spectral band characteristics of n*–*π* and π–π* were attained, and the n*–*π* and π–π* transitions were attributable to the existence of the C=O group and the aromatic ring, respectively. Assignments of n–π* and π–π* transitions in the UV-vis spectra for all the molecules are also presented in tabular form below (Table 1).

A bathochromic shift can be observed in the absorbance maxima when the solvent polarity is changed from hexane to chloroform to acetone. For OHNH, ONBr, ONCl and ONMe, a modest blue shift in the absorption maximum was observed for ethanol. These significant shifts can only be assigned to hydrogen bonding between the solute and solvent molecules. Hydrogen bond formation was quite feasible for fluorophores containing C=O groups in more polar protic solvents. In hydroxylic solvents, hydrogen bonding is crucial for defining the blue-shifted n → π * transition.^26,27^ Carbonyls in hydroxylic solvents undergo n → π* transitions that excite a stable hydrogen-bonded complex (in the ground state). Because of the removal of one electron from the n-orbital, it is expected that the complex should be destabilized in the excited state. The extent of destabilization is dependent on the extent of the decrease in dipole moments during the transition.

**Fig. 3:**
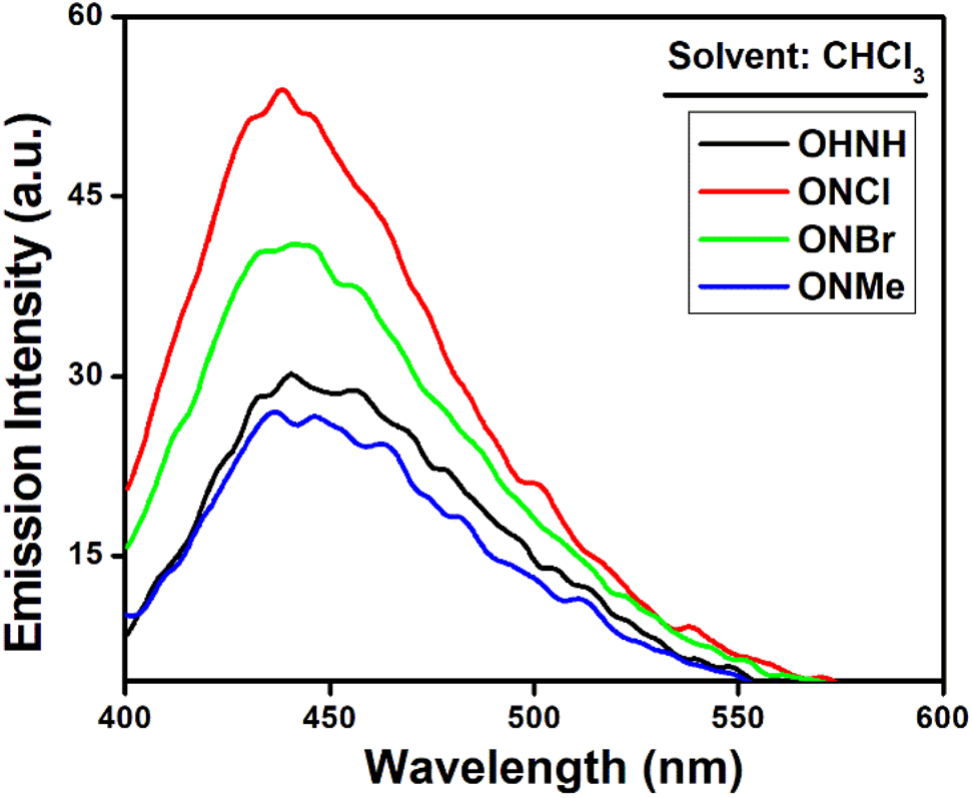
Emission spectra of OHNH, ONMe, ONCl, and ONBr in chloroform.

We examined the individual emission spectra of each chalcone derivative in various solvents due to these intriguing absorption spectrum responses (Fig. 3, see ESI†). All the chalcone derivatives in CHCl_3_ exhibited emission maxima around 440 nm under 380 nm excitation (Fig. 3). We assessed the relative quantum yields of the compounds in CHCl_3_ in order to quantify the fluorescence characteristics. For OHNH, ONMe, ONCl, and ONBr, the estimated relative quantum yields were 0.21, 0.19, 0.29, and 0.25, respectively. When analyzing fluorescence spectra, it is essential to determine whether any contamination or light scattering affects the observed red-edge effect or not. To cross-validate the results, we studied the emission spectra of the blank solvent (chloroform, ethanol, acetone and hexane) and the relative fluorescence quantum yield. Through careful observations, we confirmed that the observed bands were due to emission from the synthesized probe molecules and were not due to instrumental artifacts.

### 3.2 Tunable photoluminescence Study

**Fig. 4:**
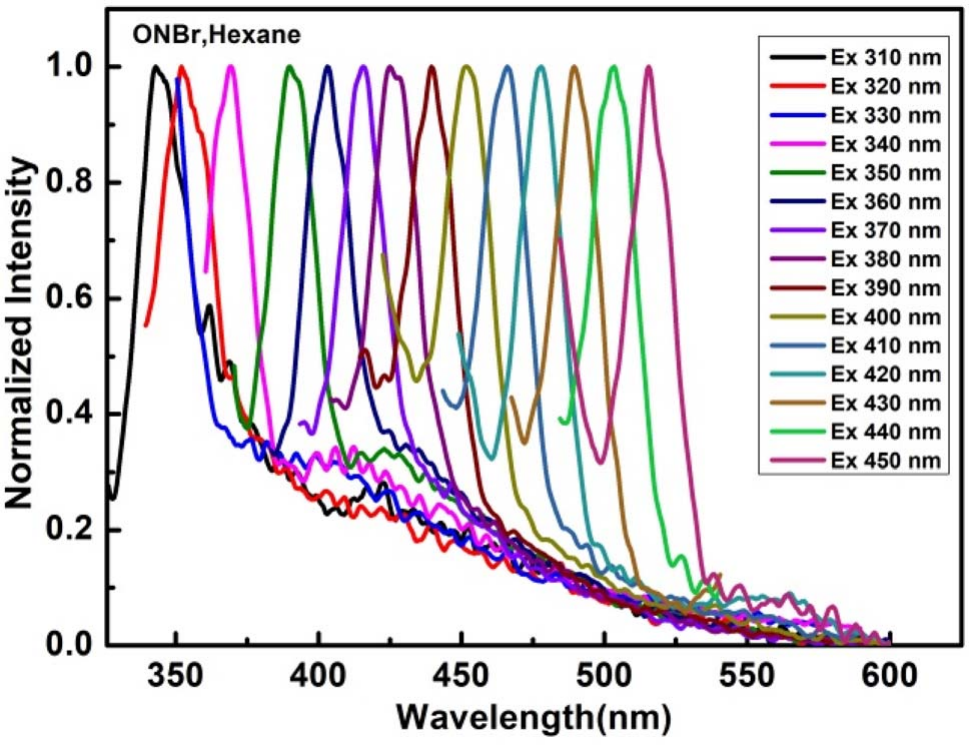
Excitation-dependent fluorescence with a continuous red shift for ONBr in hexane.

In order to determine any change in the emission spectra, the excitation wavelength was finally tuned from 310 to 450 nm at intervals of 10 nm (Fig. 4 and S3.2–S3.16†).

Excitation-dependent fluorescence [EDF] spectra were studied by sequentially altering the excitation wavelengths in hexane, chloroform, acetone and ethanol. All compounds under study in the mentioned solvent exhibited solvatochromic fluorescence when excited with 310–450 nm radiation. It was observed that (Fig. 4 and S3.2–S3.16†), with the increase in excitation wavelength at 10 nm intervals, the emission wavelength increased correspondingly for the compounds under study. This effect is generally attributed to the Red-edge effect.^28^ In hexane, all the compounds under investigation exhibited narrow emission bands (Fig. 4). However, in other polar solvents such as ethanol and acetone, broader bands were observed (see ESI†). Because there were no inhomogeneous spectra broadening in nonpolar solvents, the bands were narrower in hexane.^29^

**Fig. 5:**
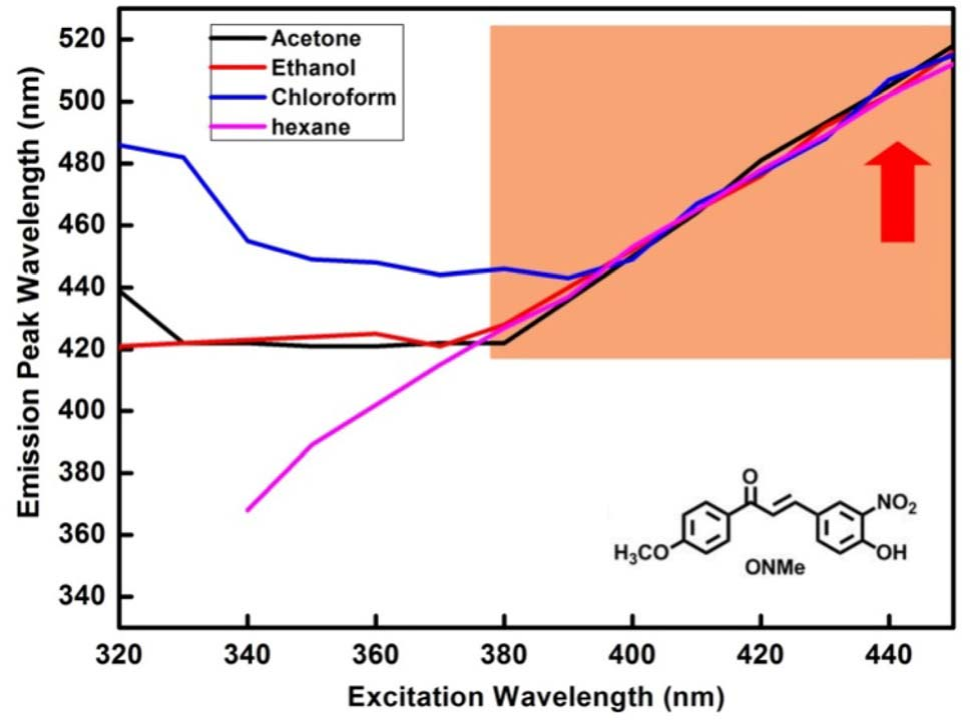
Emission *vs.* excitation plots of ONMe in different solvents.

The emission was dependent on the excitation wavelength, *i.e.*, it violated Kasha's rule. This exclusive property can be clarified by an understanding of fluorescence, which is an arbitrary event, where a fluorophore emits at various times and the rate of decay is the average of the group. Fig. 4 shows the EDF in hexane for ONBr; the plot for other compounds is shown in the ESI,† and Fig. 5 shows the emission *vs.* excitation plot. The relative quantum yield of ONBr in hexane was found to be 0.27.

The continuous spectral shift showed no change in spectral shape, and the emissions that occurred during solvent relaxation represented an average of the partially relaxed emission. This shift occurs due to emissions from an unrelaxed state. Gradually, more molecules were relaxed, resulting in longer wavelength emission. At intermediate times, emissions from both species were observed with either a blue or red shift. The redshift observed at around 370 nm excitation has a solvent configuration similar to that of the relaxed state. These fluorophores are usually more strongly hydrogen bonded to the solvent, so a red-shifted emission is observed.

### 3.3 Computational study

**Fig. 6:**
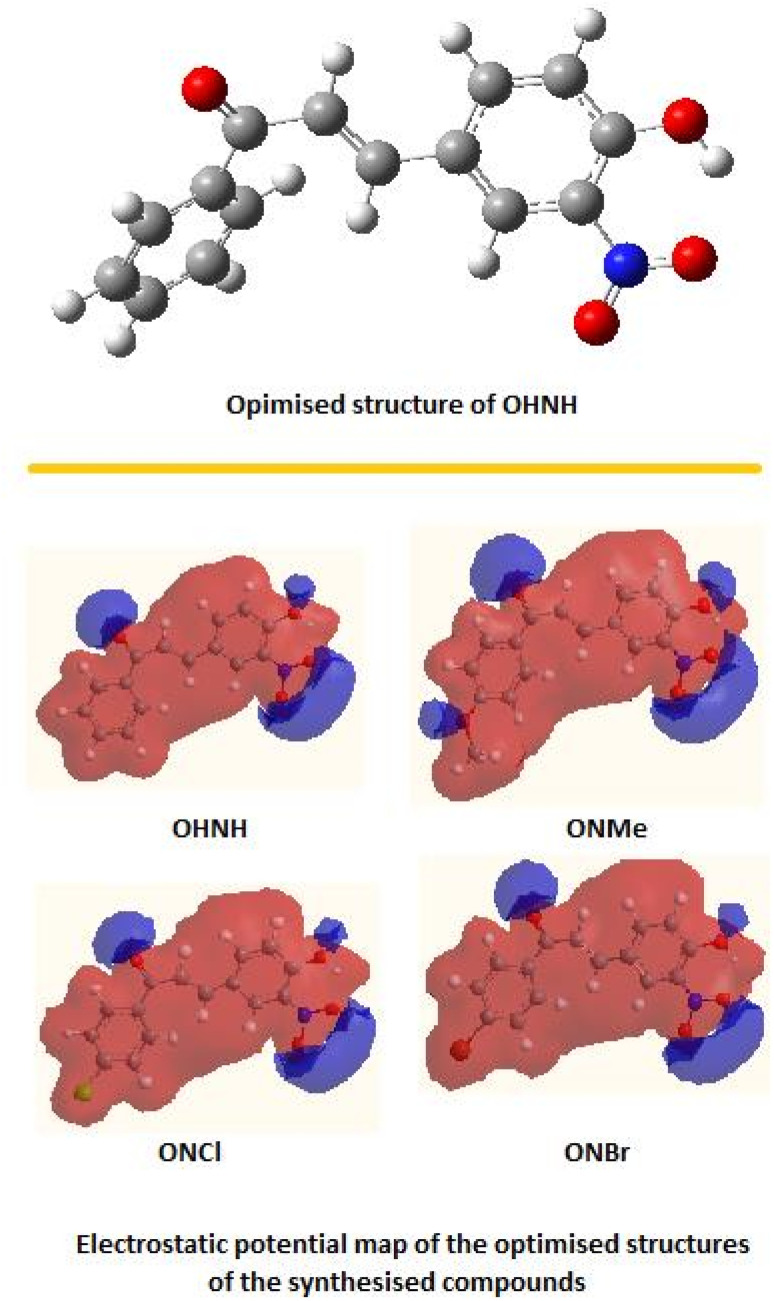
Optimized structure of OHNH and electrostatic potential map of the studied four compounds.

Computational analysis of all synthesized molecules were performed, and optimization studies on OHNH revealed a twisted structure (Fig. 6). As previously mentioned, the GAUSSIAN 03 package in conjunction with the BRAF supercomputing environment was used to perform all computations. Using 6–31++g (d, p), the structures of OHNH, ONMe, ONCl, and ONBr were completely optimized using Becke's three-parameter hybrid exchange function in conjunction with the Lee–Yang–Parr nonlocal correlation functional (B3LYP) DFT.

**Fig. 7:**
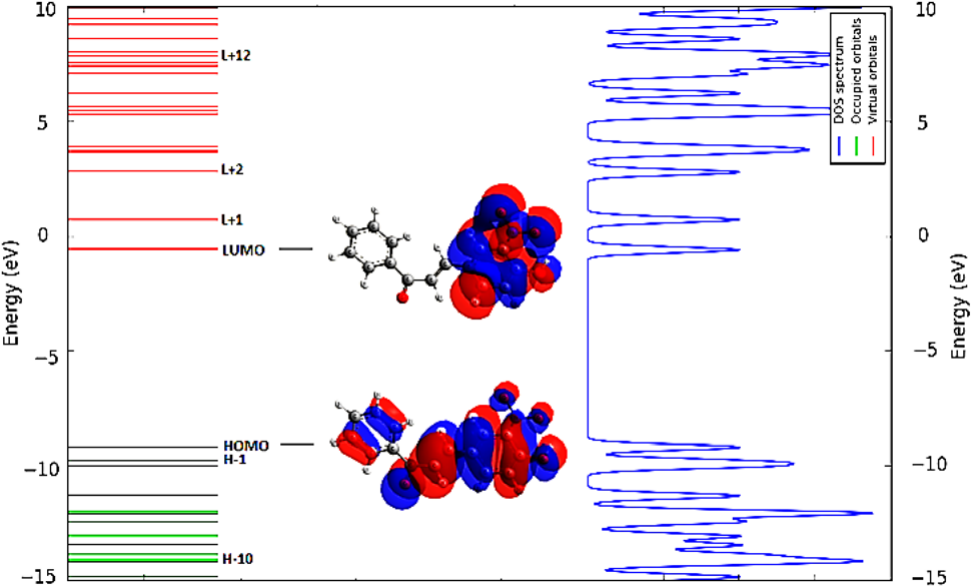
Density of state (DOS) spectrum of OHNH.

The theoretical studies on OHNH (Fig. 6) revealed that the contributors primarily responsible for the observed electronic transition were not HOMO > LUMO only but rather spanned from HOMO-10 to LUMO + 12 (Table S1 can be found in the ESI†). The Density of State (DOS) spectrum (Fig. 7) of this compound indicates the presence of very closely spaced energy levels (both filled and virtual). These intimate energy levels may induce environmental sensitivity in the compound.

The closely spaced energy levels of these compounds probably increase the possibility of transitioning to higher energy levels. The compactly spaced electronic states allow the transiting electron to “always” access a suitable energy level. Consequently, fluorescence activities and flexible light-emitting properties are imparted by the compounds.

### 3.4 Biosensing with tunable photoluminescence

With this versatile tunable photoluminescence property (EDF), we were quite fascinated to explore its application as a biosensor. With such an intension we have studied their application as biosensors for murine hepatocytes. As described here, the reported approach^30,31^ was used to isolate murine hepatocytes. For fluorescence microscopy, murine hepatocytes were isolated by the method of R Prajapati and R Patel mainly.^30^ In brief, the dissected murine liver was macerated with Alsever's solution and then digested in 0.5% trypsin–EDTA for 30 minutes with occasional aspiration. The lysate was allowed to settle for debris in ice for one hour. The upper single cell suspension was centrifuged at 800 g, washed twice with DPBS and finally resuspended with 10^6^ cells per ml. 100 microliters of the cell suspension was incubated with the compounds for 15 minutes and washed thrice with DPBS, smeared on a glass slide and observed under a fluorescent microscope (Nikon ECLIPSE TS100) with an absorption filter 361–389 and emission filter 430–490.

**Fig. 8:**
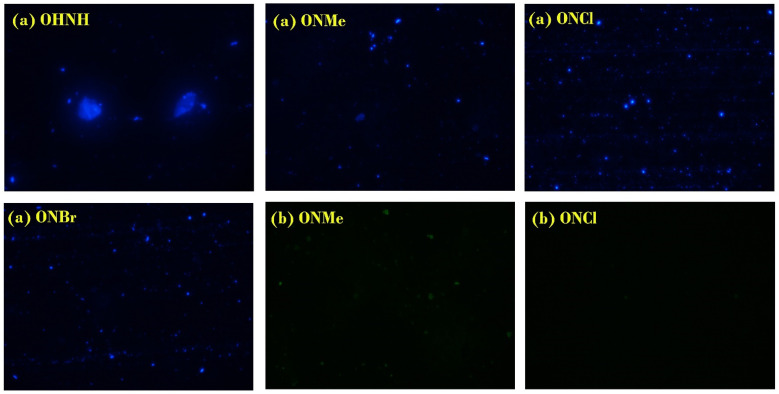
Fluorescence micrographs of OHNH (a), ONMe (a), ONCl (a) and ONBr (a) show the blue micrograph when excited with 361–389 nm UV light. ONMe (b) and ONCl (b) show the green micrograph when excited with 430–490 nm UV light.

Synthesized Donor–π–Acceptor chalcones displayed color-tunable emission properties and were truly harmonized when these compounds were subjected to sensing by hepatocyte cells. The hepatocytes were visualized in the fluorescent image under UV irradiation at 361–389 nm and 430–490 nm. Interestingly, 400× image showed endosomes with brilliant blue color when irradiated with 361–389 nm for all probes, but ONMe and ONCl also stained them green when excited at 430–490 nm (Fig. 8; ONMe (b), ONCl (b)). As targeted, by varying the wavelength, the fluorescent emission also changed. The compound at 0.1 × 10^−10^ M concentration was most likely impermeable to the living cells and entered the cells through endocytosis, as evident from the marginalized endosomes, as seen in the micrograph (Fig. 8).

## 4. Conclusion

In this study, we demonstrate that excitation-tunable emitters can be produced through a single, one-step chemical synthesis. Their characteristic distinctive photoluminescence spectra in different solvents were studied. It was intriguing to examine how the compounds responded to the solvent characteristics, as evidenced by the narrow bands in the emissions and blue shift. In the instance of EDF, the design approach was successful for all four molecules, with the brightest OHNH sensing up at 361–389 nm. However, only ONCl and ONMe could tune their sensing property at 430–490 nm also. These observations indicate that the –OMe group was more active in the chosen design and used in the study, followed by the –Cl group. Our new fluorescent chromophore opens new possibilities for the application of excitation-dependent sensing and is a valuable tool for the detection of receptors in cells and tissues. It is anticipated that research using these novel chromophores in various tissues will further demonstrate their use as pharmacological tools.

## Data availability

Data will be available from authors on justified request.

## Conflicts of interest

The authors declare that they have no conflict of interest.

## Supplementary Material

RA-015-D4RA06978A-s001
