# Supplementary material for: Red edge effect of chalcone derivatives and their application in Bio-sensing
Source: RSC Adv. 2025 Apr 28;15(17):13505–12. doi: 10.1039/d4ra06978a (PMC12035810; doi:10.1039/d4ra06978a)
Supplement: RA-015-D4RA06978A-s001 [file RA-015-D4RA06978A-s001.pdf]

Supplementary Information (SI) for RSC Advances.
This journal is © The Royal Society of Chemistry 2025

Red Edge effect of chalcone derivatives and their application in biosensing

Amrita Saha[a,b*], Monaj Karar[a*], Sudip Choudhury[b]

[a] *MLR Institute of Technology, Hyderabad, Telangana 500043, India.*

[b]*Department of Chemistry, Assam University, Silchar, Silchar-788 011*

*Email: drmonajkarar@mlrinstitutions.ac.in, dramrita@mlrinstitutions.ac.in

Keywords: *Chalcone; Red Edge effect; bio-sensor, excitation-dependent emission; fluorescent probe.*

Table S1: Description of ^{1}H NMR and ^{13}C Figures presented in *S.I* 1.1-1.8

Figure	Description
1.1	^{1}H NMR spectrum of *(E)-1-(4-bromophenyl)-3-(4-hydroxy-3-nitrophenyl) prop-2-en-1-one* ONBr
1.2	^{13}C NMR spectrum of *(E)-1-(4-bromophenyl)-3-(4-hydroxy-3-nitrophenyl) prop-2-en-1-one* ONBr
1.3	^{1}H NMR spectrum of *(E)-1-(4-bromophenyl)-3-(4-hydroxy-3-nitrophenyl) prop-2-en-1-one* ONCl
1.4	^{13}C NMR spectrum of *(E)-1-(4-bromophenyl)-3-(4-hydroxy-3-nitrophenyl) prop-2-en-1-one* ONCl
1.5	^{1}H NMR spectrum of *(E)-1-(4-bromophenyl)-3-(4-hydroxy-3-nitrophenyl) prop-2-en-1-one* ONMe
1.6	^{13}C NMR spectrum of *(E)-1-(4-bromophenyl)-3-(4-hydroxy-3-nitrophenyl) prop-2-en-1-one* ONMe
1.7	^{1}H NMR spectrum of *(E)-1-(4-bromophenyl)-3-(4-hydroxy-3-nitrophenyl) prop-2-en-1-one* OHNH.

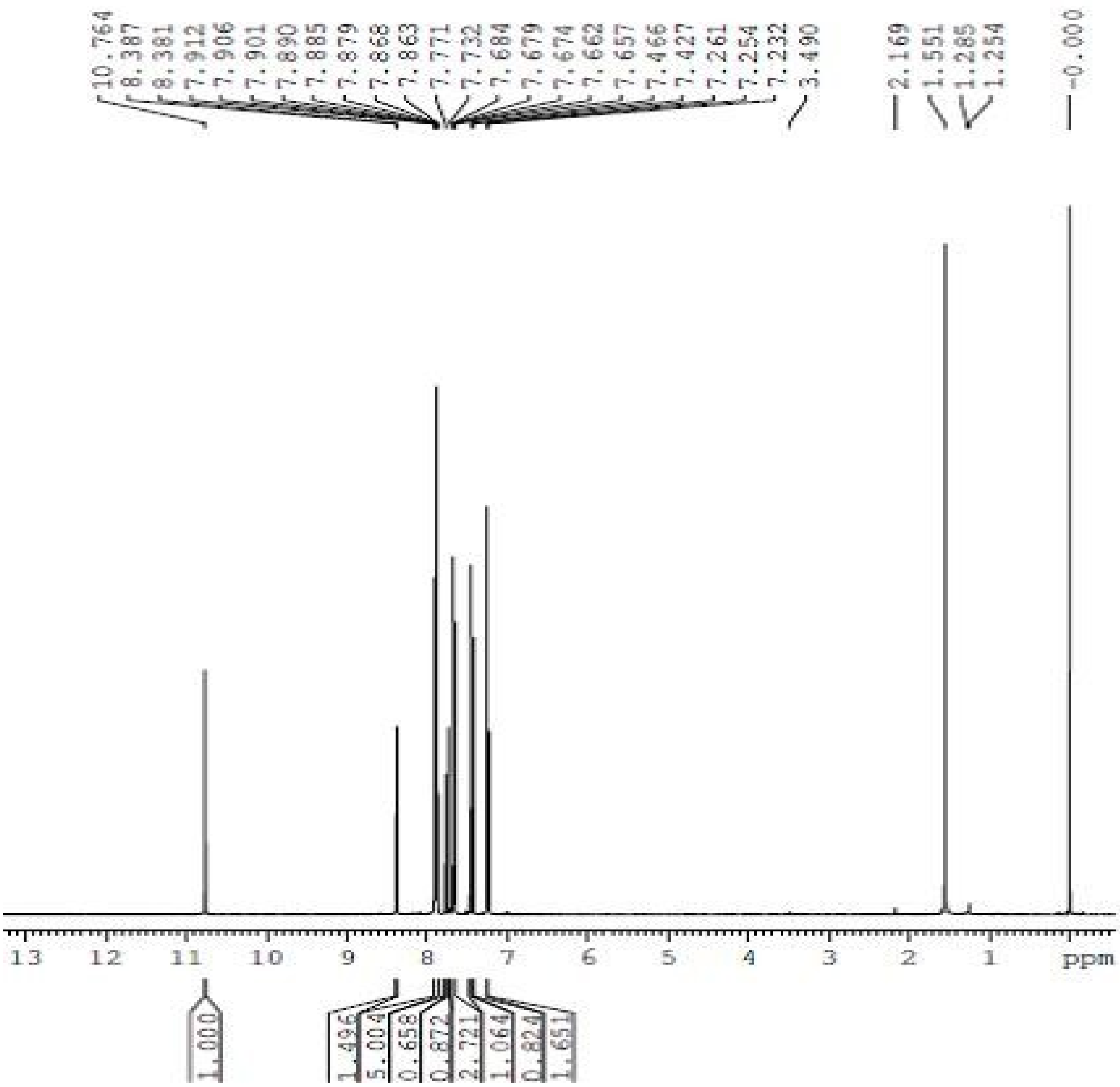

Figure.1.1: ^{1}H NMR spectrum of *(E)-1-(4-bromophenyl)-3-(4-hydroxy-3-nitrophenyl) prop-2-en-1-one* ONBr

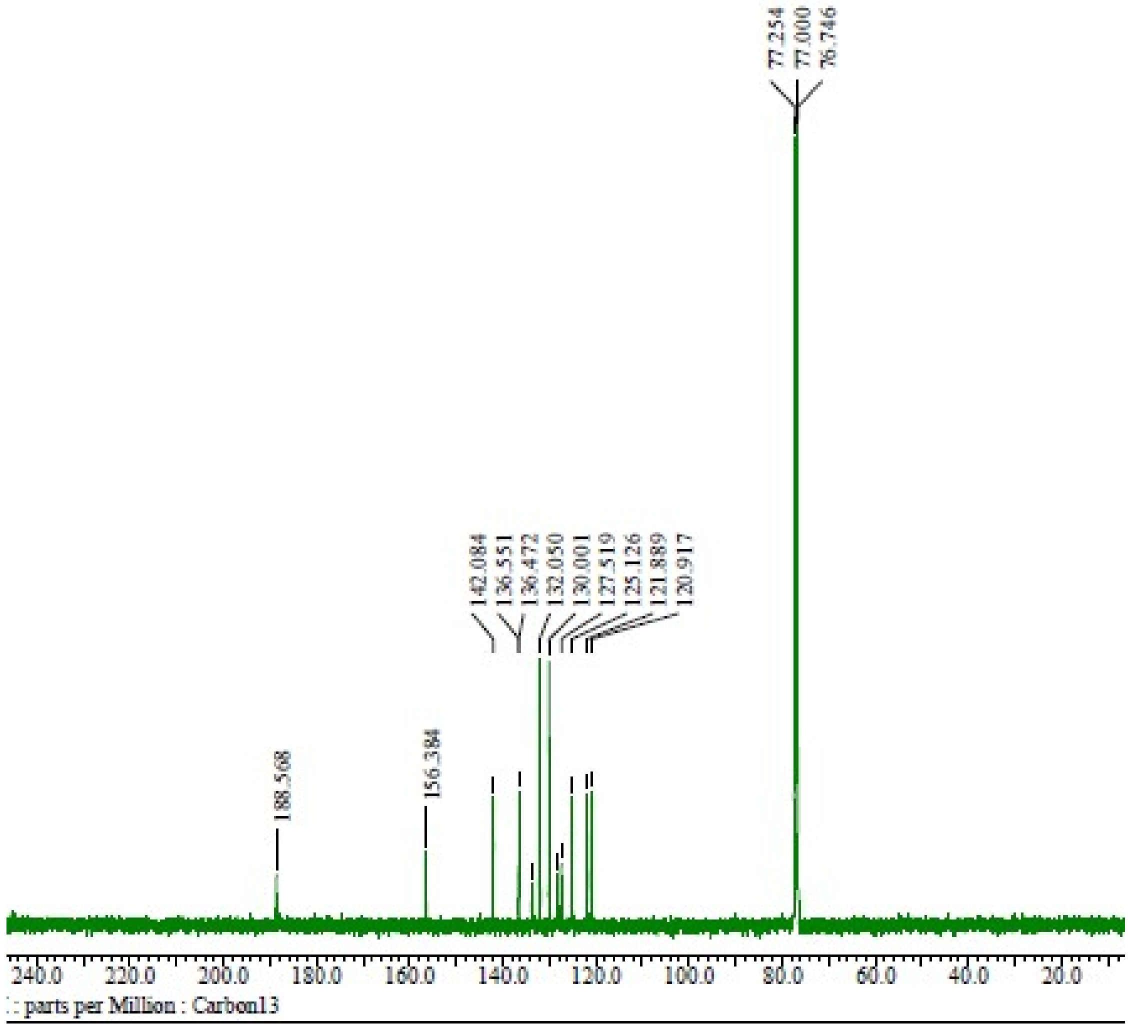

Figure 1.2: ^{13}C NMR spectrum of *(E)-1-(4-bromophenyl)-3-(4-hydroxy-3-nitrophenyl) prop-2-en-1-one* ONBr

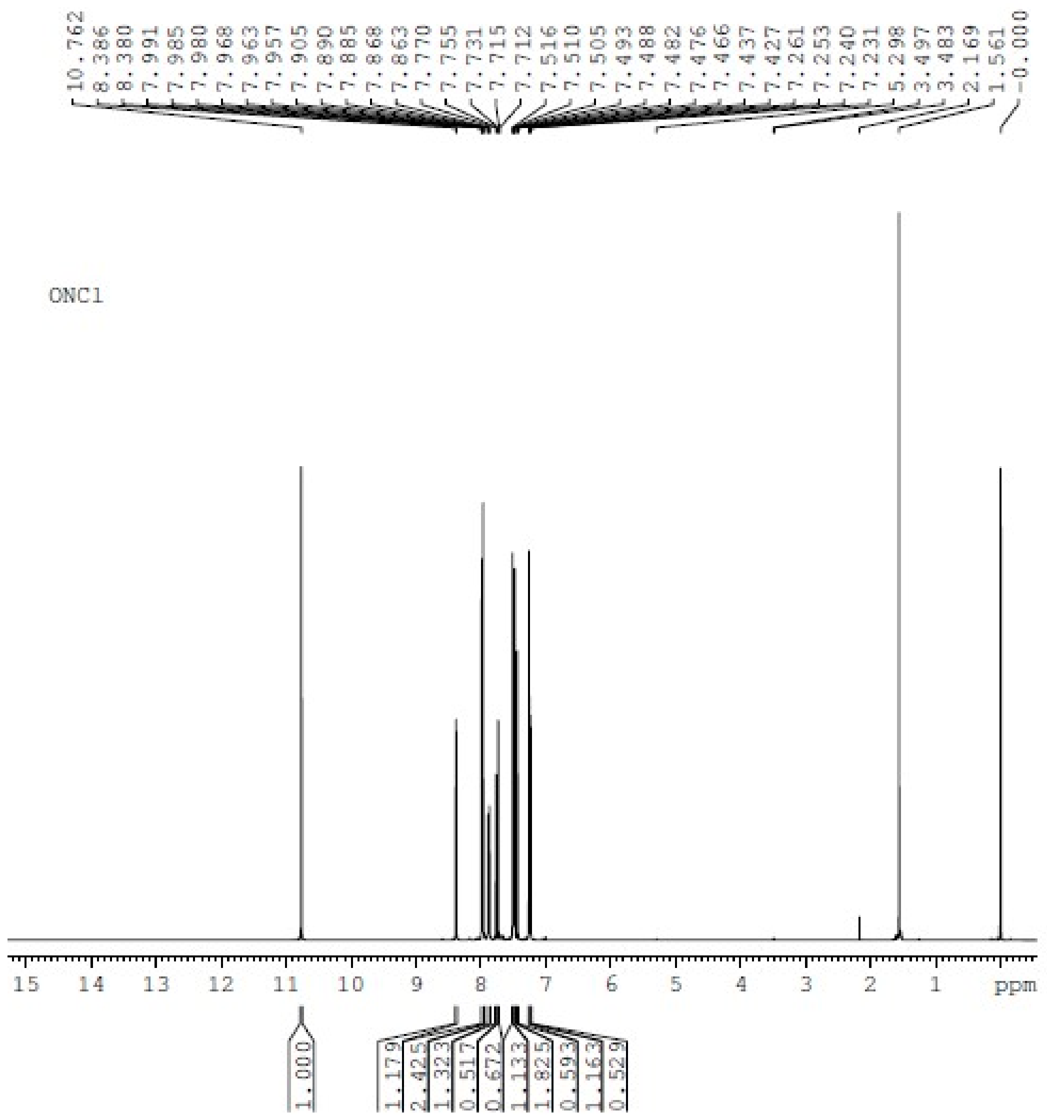

Figure 1.3: ^{1}H NMR spectrum of *(E)-1-(4-chlorophenyl)-3-(4-hydroxy-3-nitrophenyl) prop-2-en-1-one* ONCl

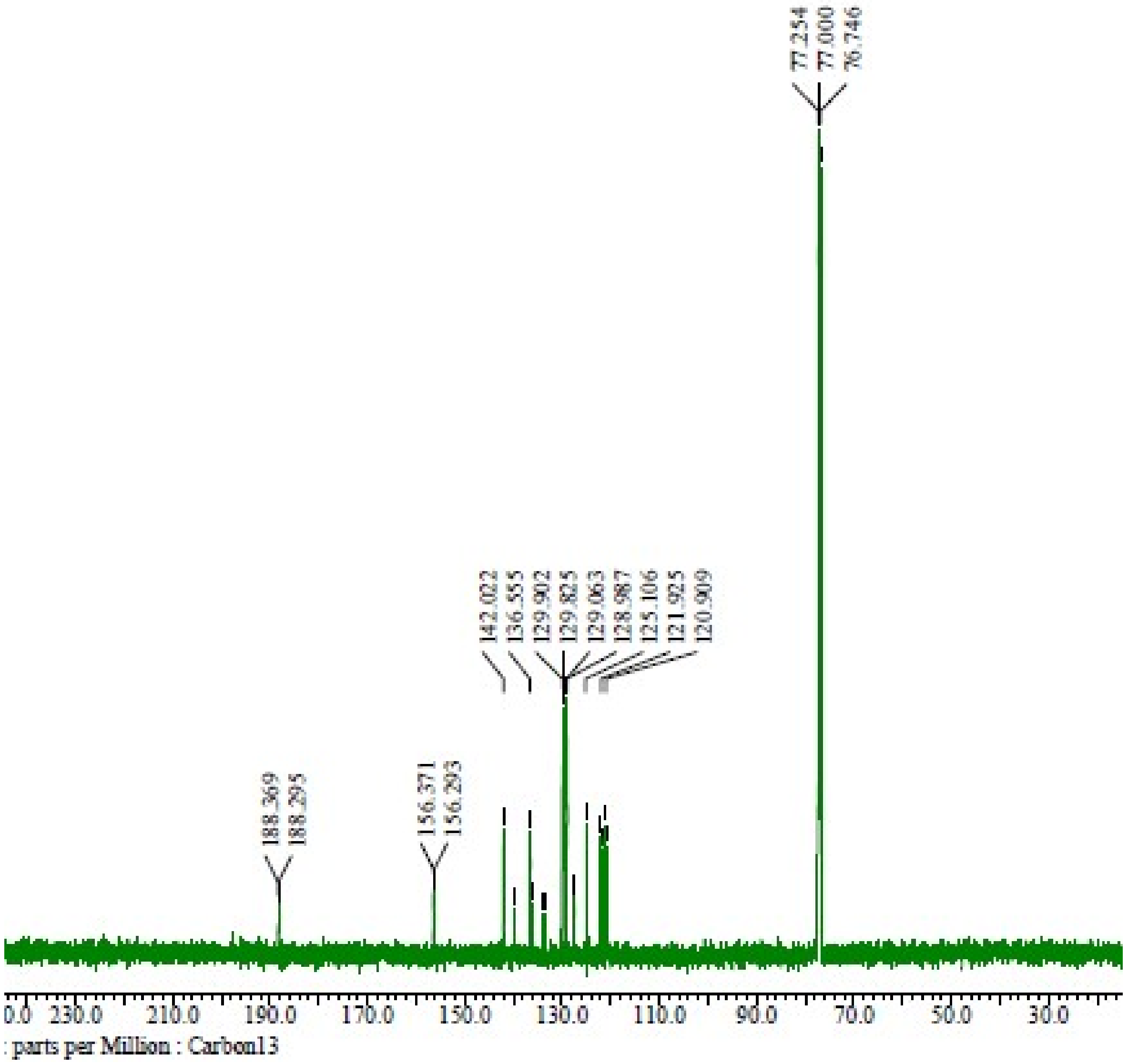

Figure 1.4: ^{13}C NMR spectrum of *(E)-1-(4-chlorophenyl)-3-(4-hydroxy-3-nitrophenyl) prop-2-en-1-one* ONCl

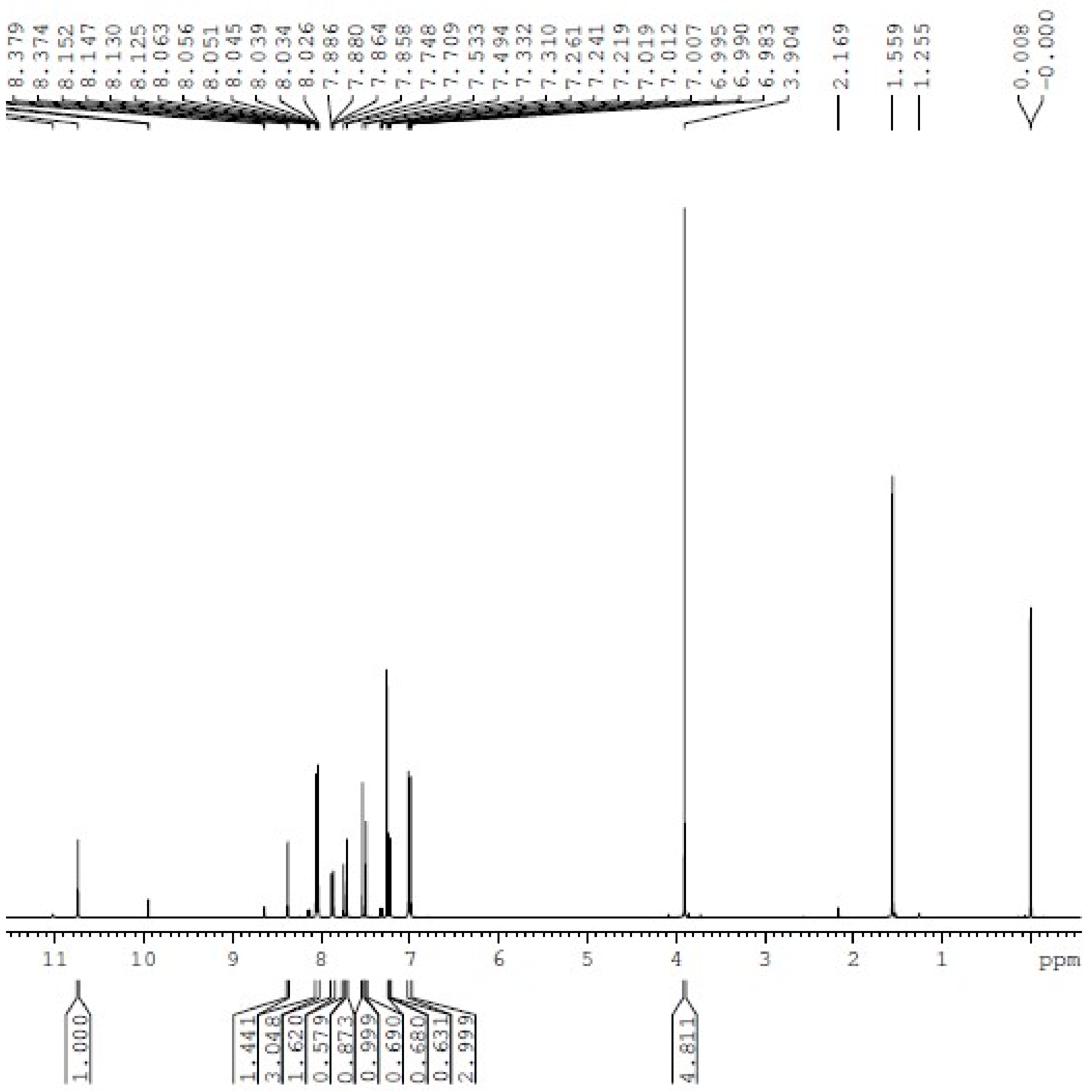

Figure S1.5: ^{1}H NMR spectrum of *(E)-3-(4-hydroxy-3-nitrophenyl)-1-(4-methoxyphenyl)prop-2-en-1-one* ONMe

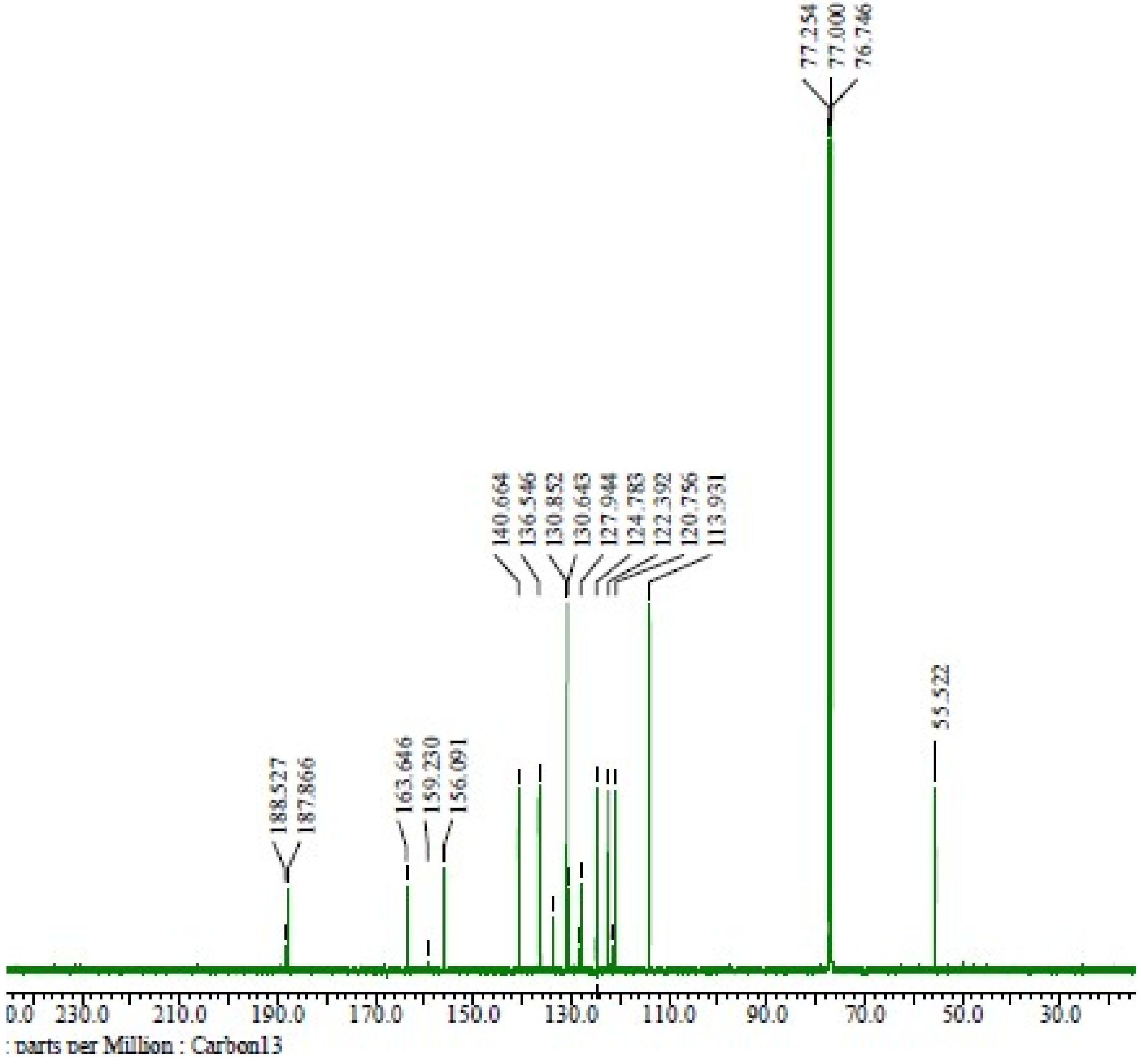

Figure 1.6: ^{13}C NMR spectrum of ***(E)-3-(4-hydroxy-3-nitrophenyl)-1-(4-methoxyphenyl)prop-2-en-1-one*** **ONMe**

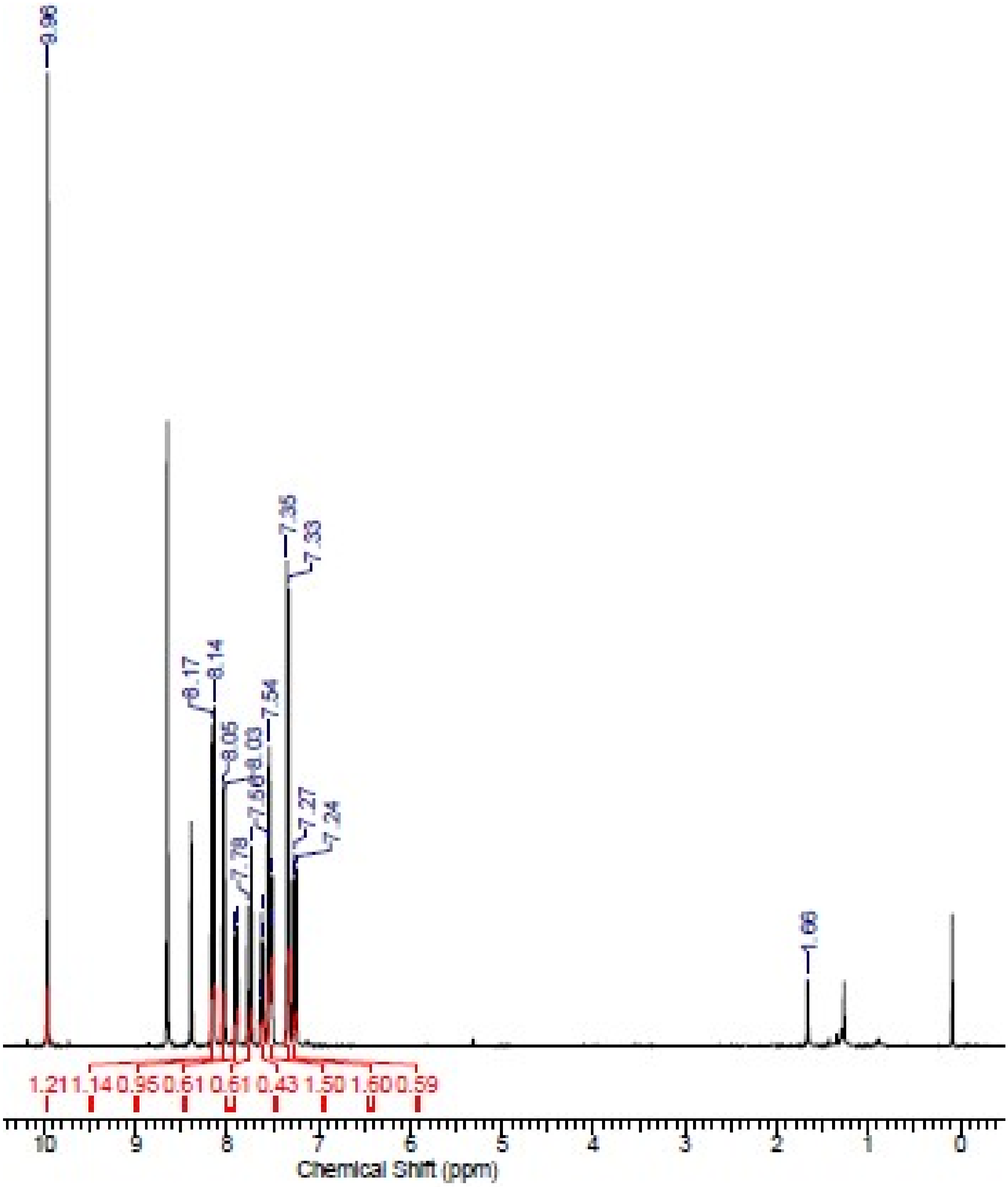

Figure 1.7: ^{1}H NMR spectrum of *(E)-3-(4-hydroxy-3-nitrophenyl)-1-phenylprop-2-en-1-one* OHNH

Table S2: Description of Absorption Spectra presented in Figures S.I 2.1-2.4

Figure	Description
211	Absorption spectra of ONBr in different solvent.

2.2	Absorption spectra of ONCl in different solvent.
2.3	Absorption spectra of ONMe in different solvent.
2.4	Absorption spectra of OHNH in different solvent.

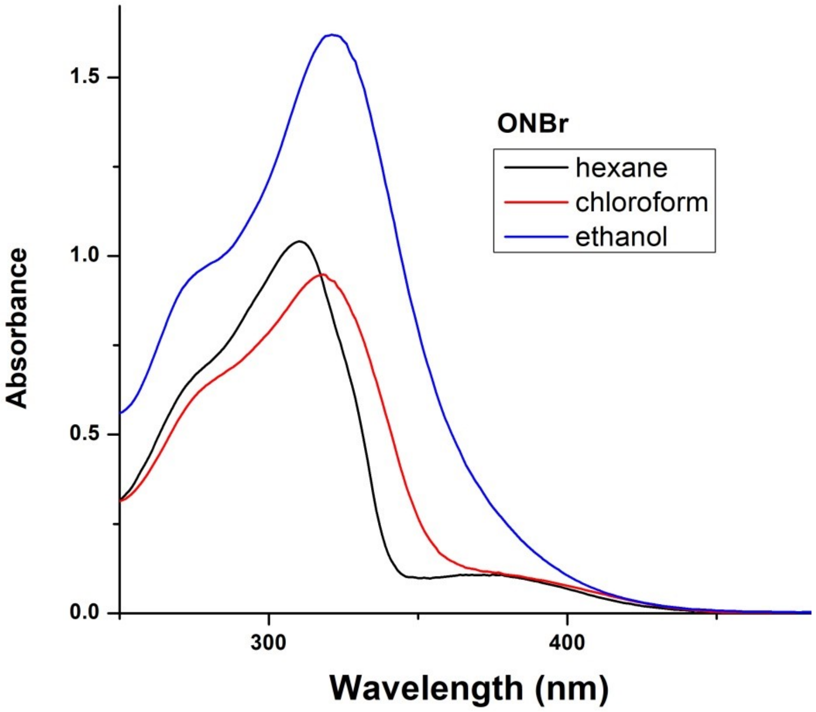

Figure S2.1: Absorption spectra of ONBr in different solvent.

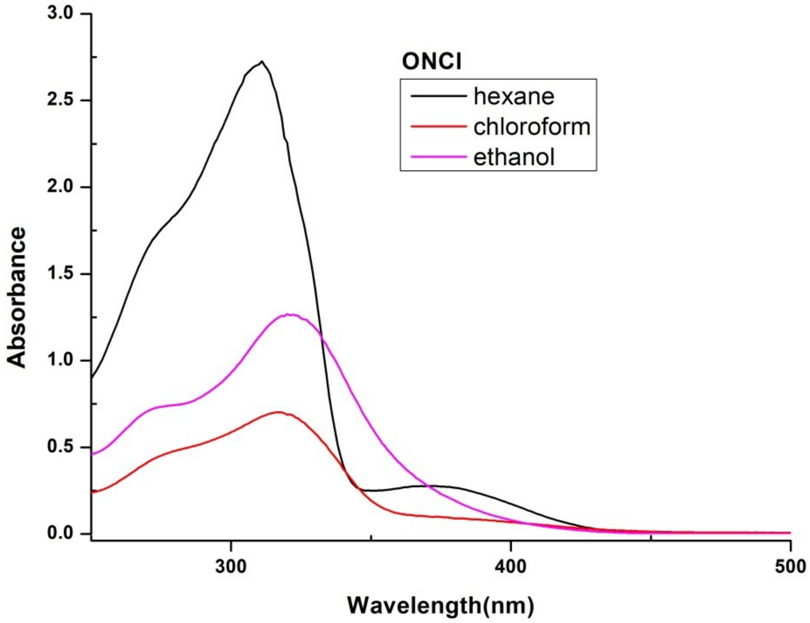

Figure S2.2: Absorption spectra of ONCl in different solvent.

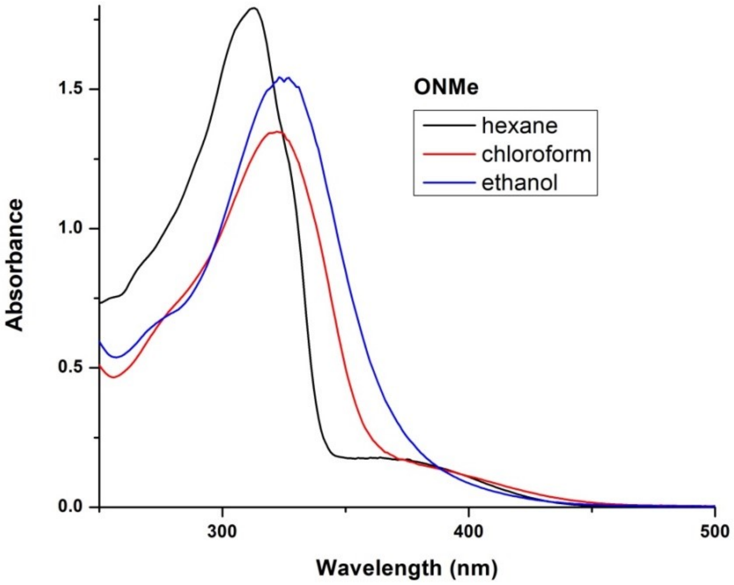

Figure S2.3: Absorption spectra of ONMe in different solvent.

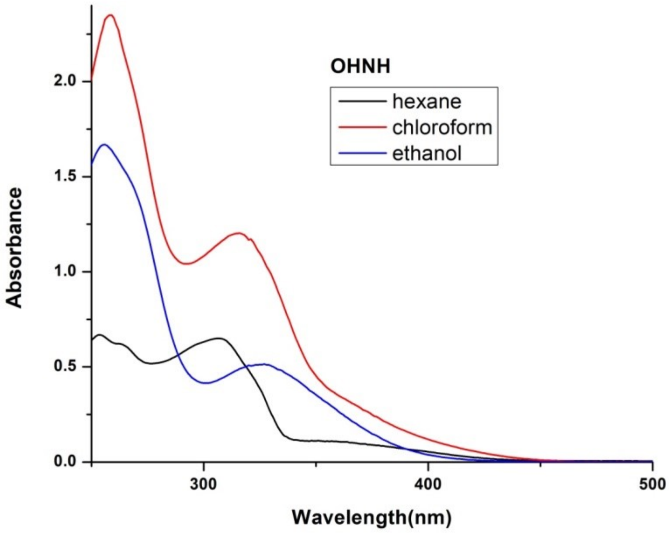

Figure S2.4: Absorption spectra of OHNH in different solvent.

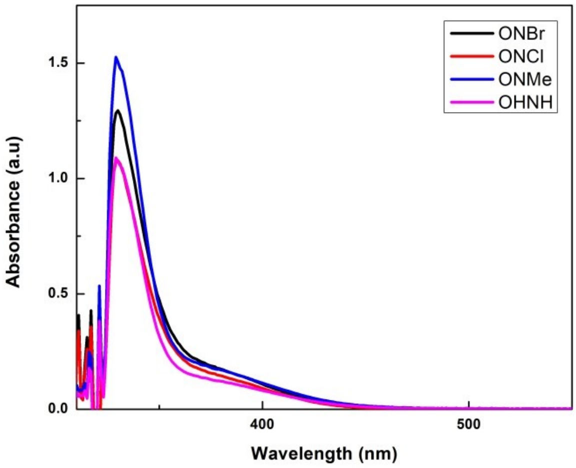

Figure 2.5: UV spectra of all compounds in Acetone

Table S3: Description of Excitation Dependent Fluorescence Spectra presented in Figures 3.1-3.

Figure	Description
	EDF of ONBr in hexane (See main article Figure2)
3.2	EDF of ONBr in Ethanol
3.3	EDF of ONBr in Chloroform
3.4	EDF of ONBr in Acetone
3.5	EDF of ONCl in hexane
3.6	EDF of ONCl in Ethanol
3.7	EDF of ONCl in Chloroform
3.8	EDF of ONCl in Acetone
3.9	EDF of ONMe in hexane
3.10	EDF of ONMe in Ethanol
3.11	EDF of ONMe in Chloroform
3.12	EDF of ONMe in Acetone
3.13	EDF of OHNH in hexane
3.14	EDF of OHNH in Ethanol
3.15	EDF of OHNH in Chloroform
3.16	EDF of OHNH in Acetone

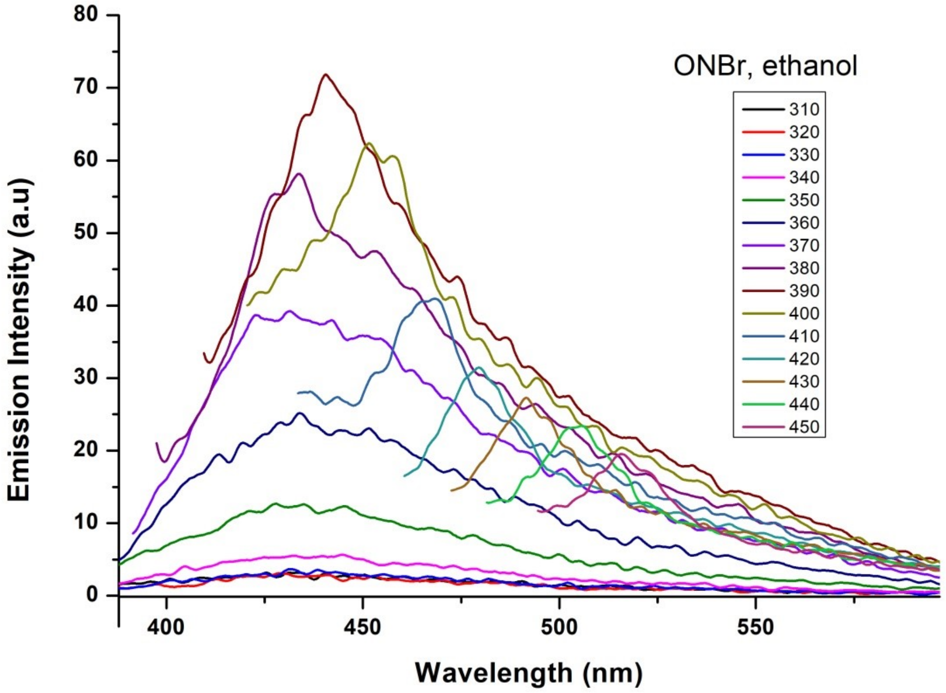

Figure S3.2: EDF of ONBr in Ethanol

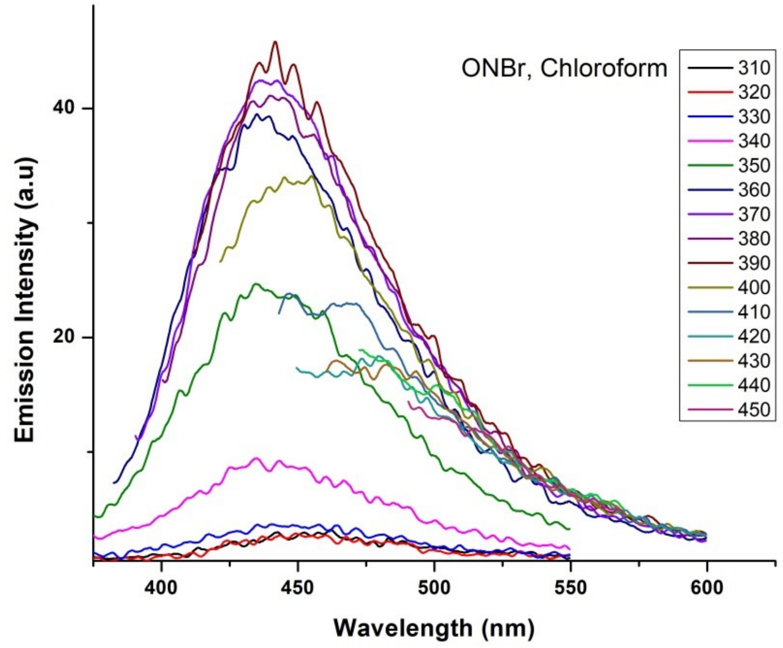

Figure S3.3: EDF of ONBr in Chloroform

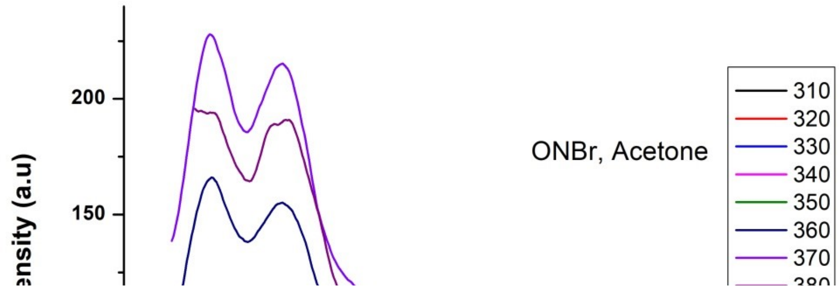

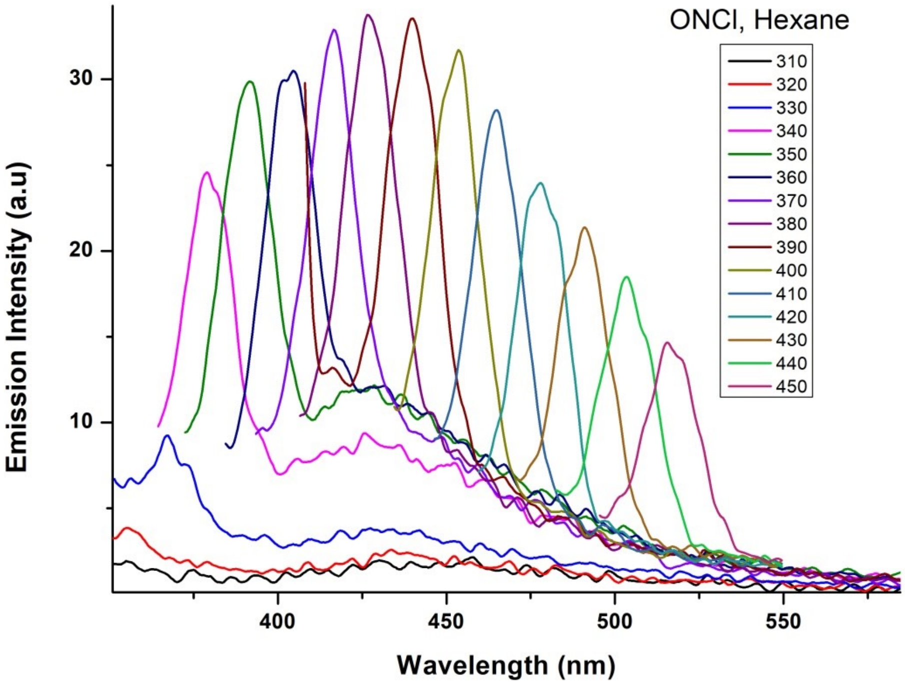

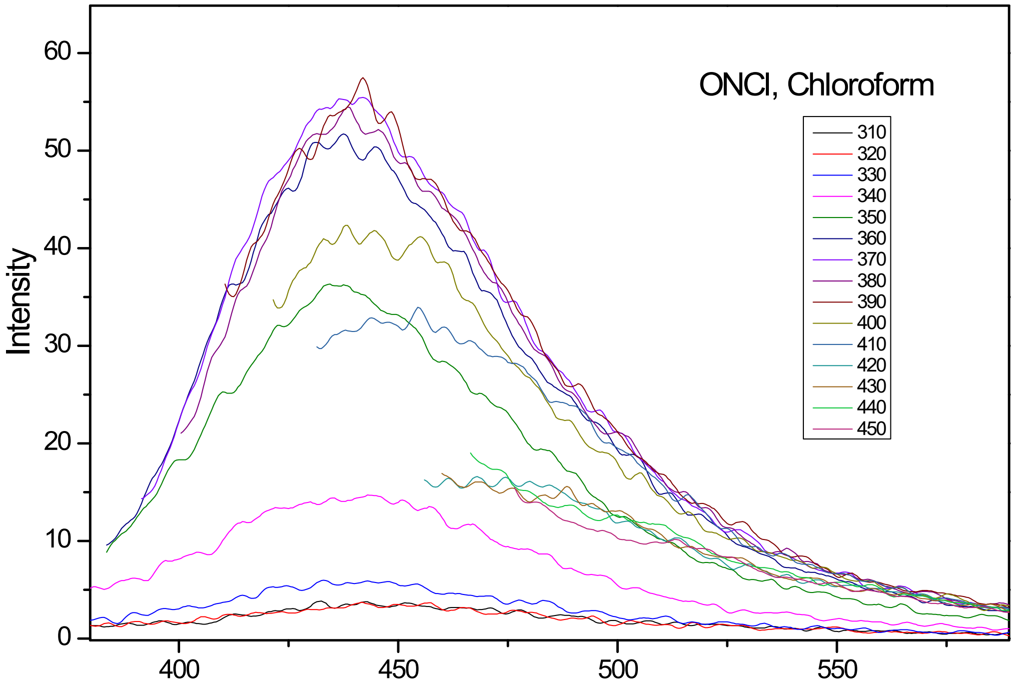

Figure S3.5: EDF of ONCl in Hexane.

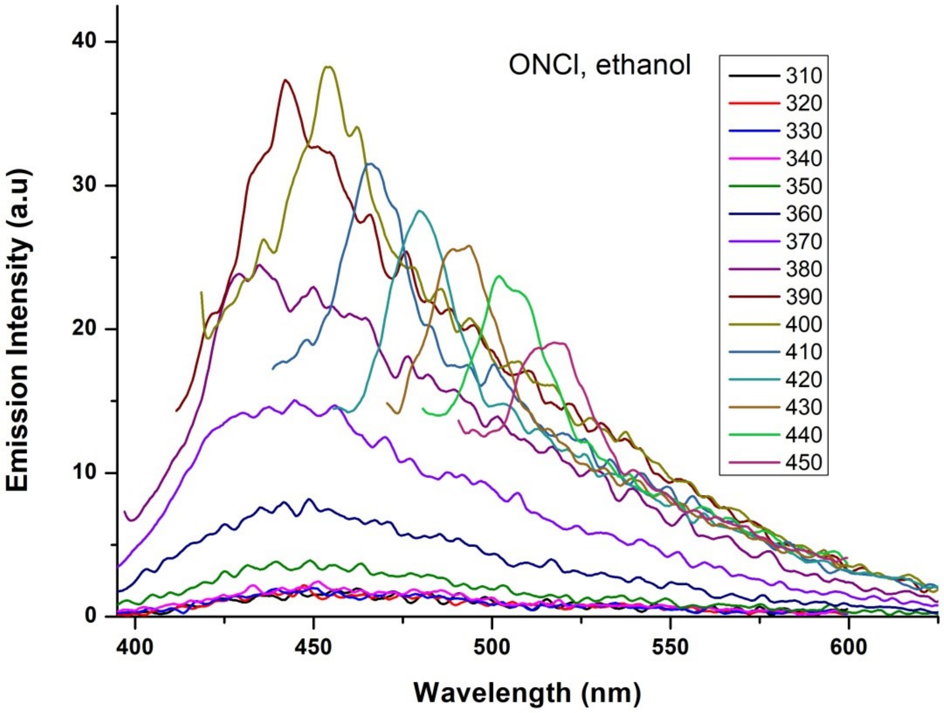

Figure S3.6: EDF of ONCl in Ethanol.

Figure 3.7: EDF of ONCl in Chloroform.

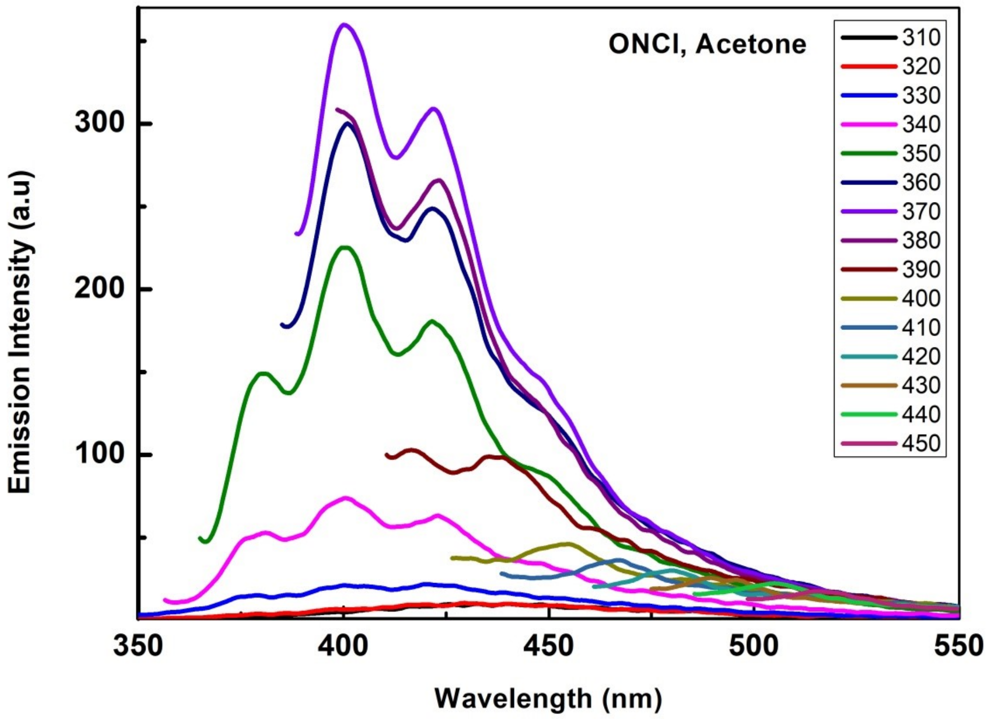

Figure S3.8: EDF of ONCl in Acetone.

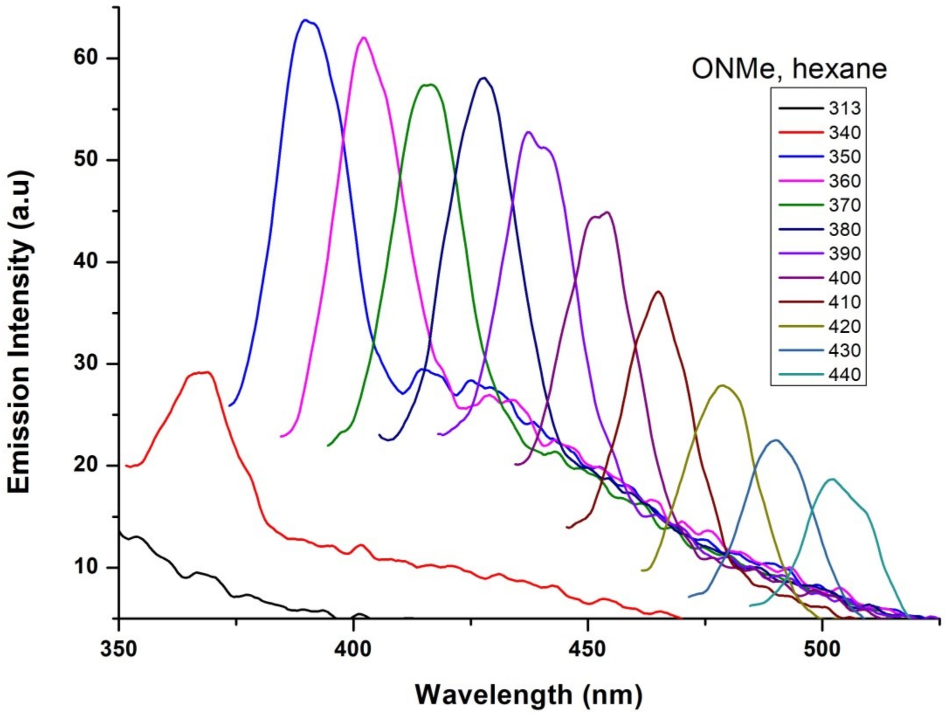

Figure S3.9: EDF of ONMe in hexane

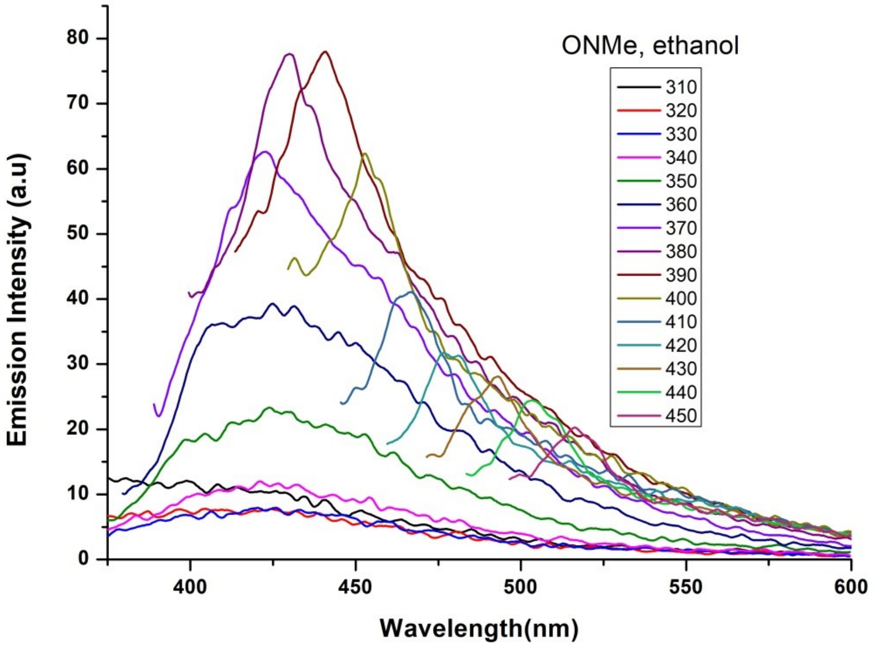

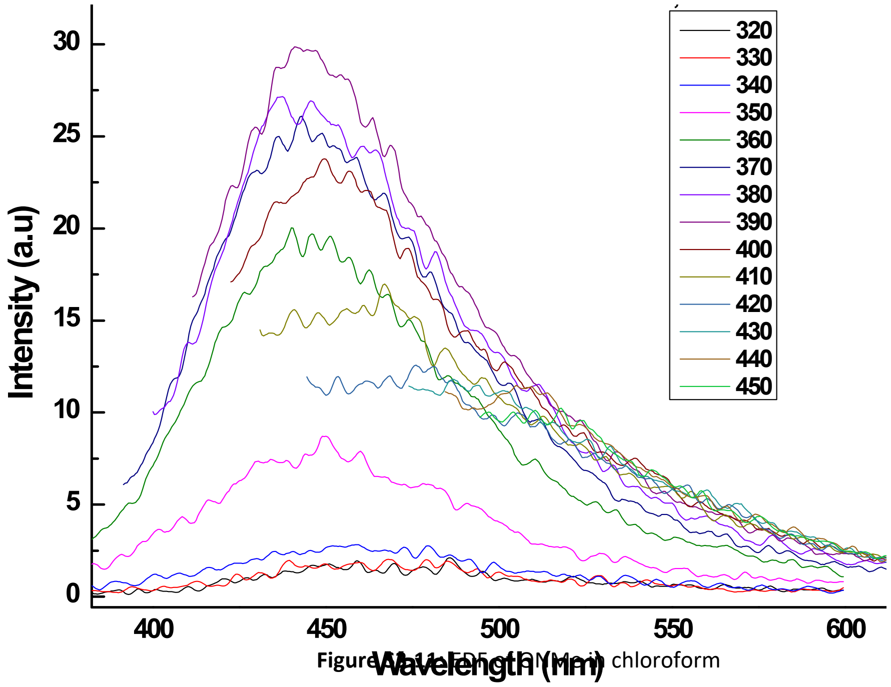

Figure [illegible]: EDF of ONMe in chloroform

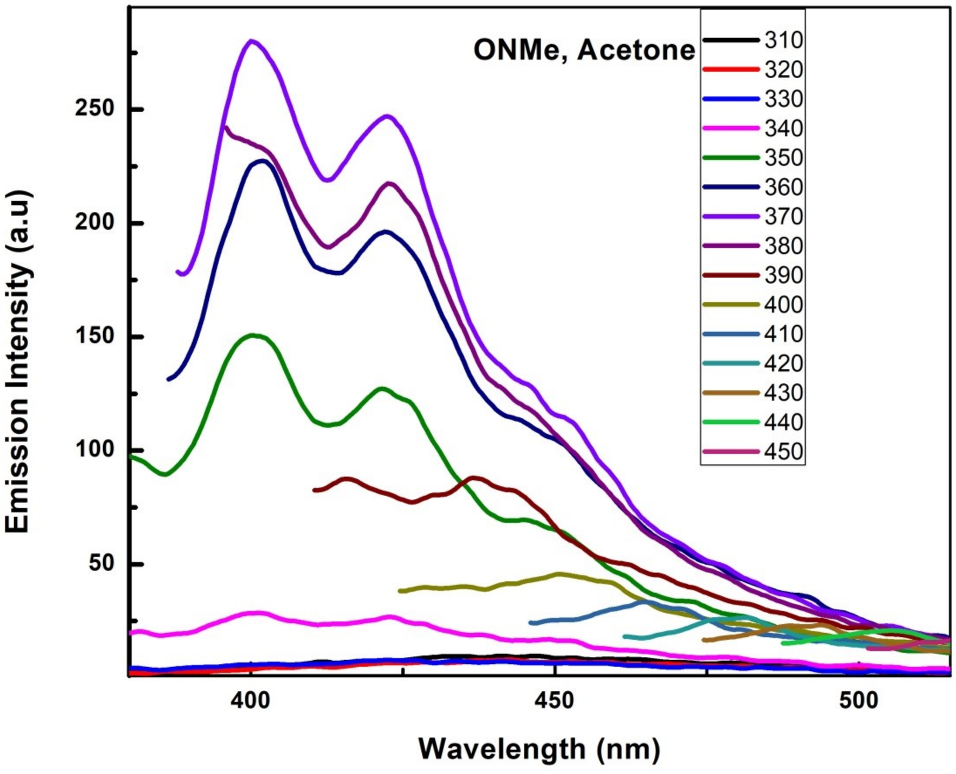

Figure S3.11: EDF of ONMe in acetone

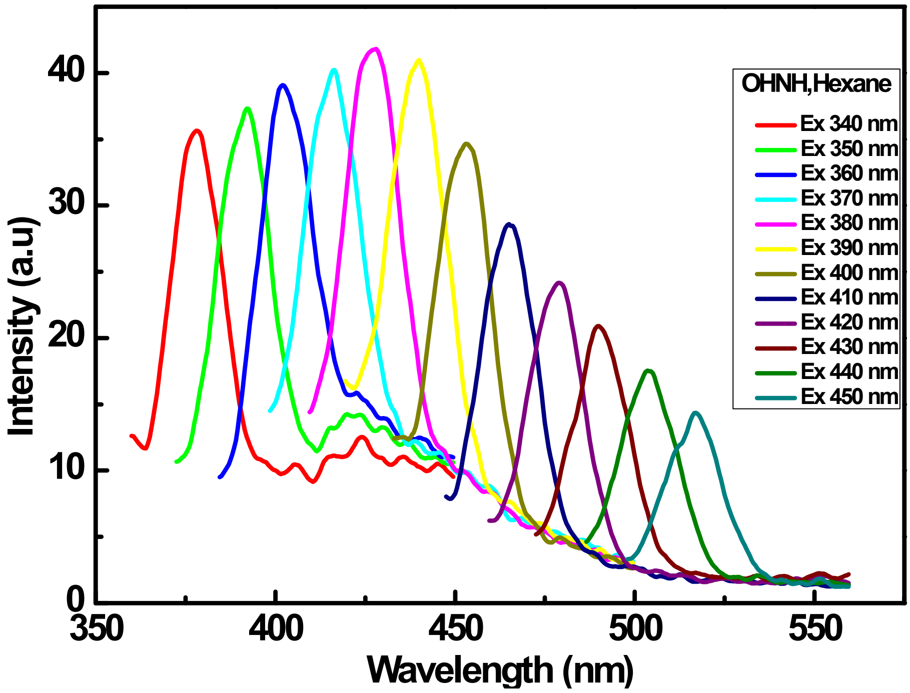

Figure S3.13: EDF of OHNH in Hexane

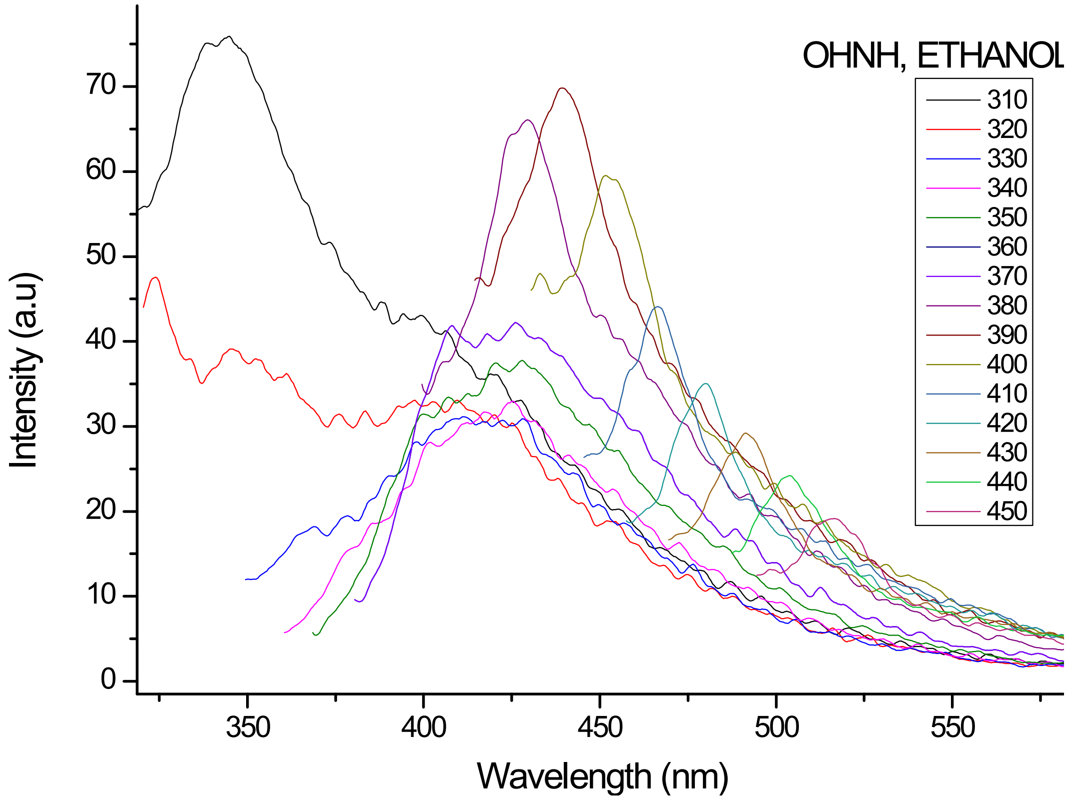

Figure S3.14: EDF of OHNH in Hexane

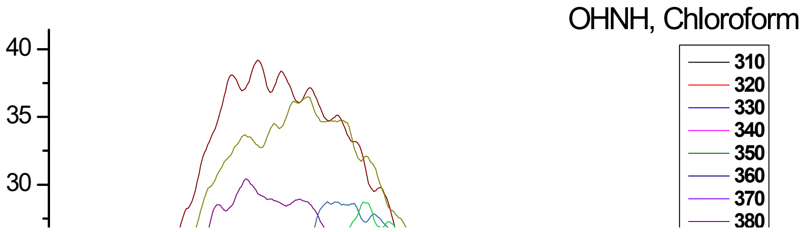

Figure S3.15: EDF of OHNH in Chloroform

Figure S3.16: EDF of OHNH in Acetone

Table S4: The calculated UV-Visible spectrum of OHNH

Entry	Absorbance maxima (nm)		Contributors
	Experimental	Theoretical	
**OHNH** (Hexane)	306	294	HOMO->LUMO (83%), H-6->LUMO (4%), H-5->LUMO (3%)
		296	H-4->L+1 (67%) H-4->L+2 (8%), H-4->L+6 (6%), H-4->L+12 (5%)
	360	351	H-7->LUMO (75%) H-10->LUMO (4%), H-7->L+3 (6%)

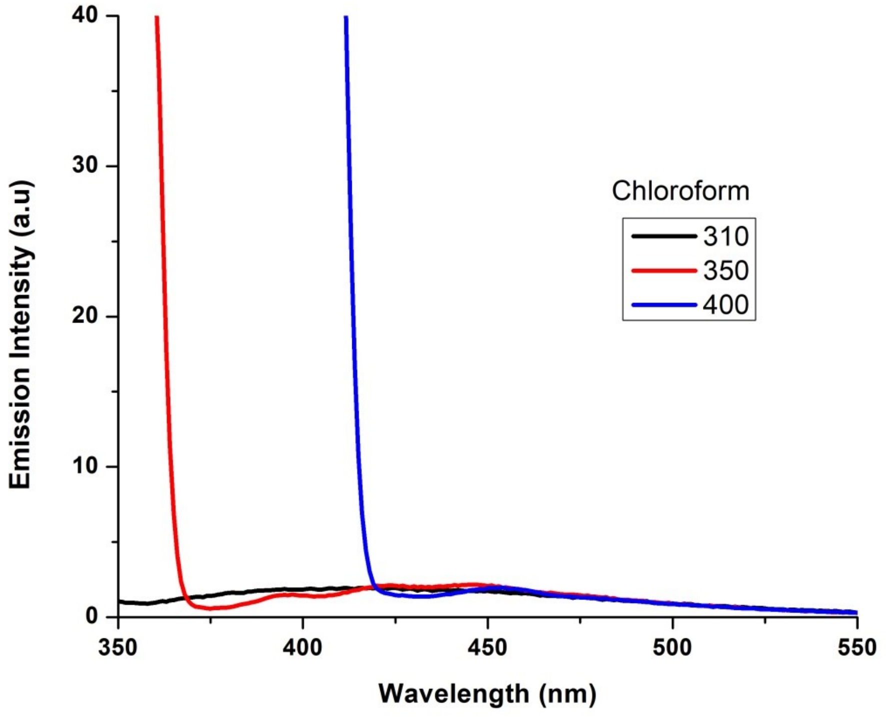

Figure S4.1: Blank Solvent spectra of Chloroform

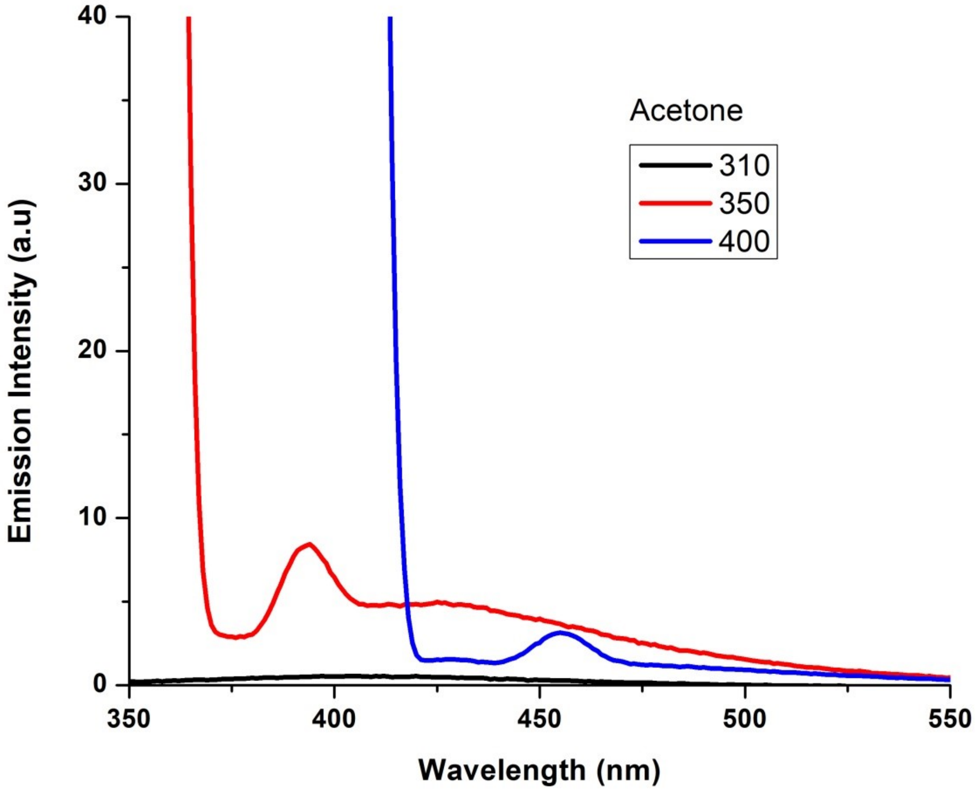

Figure S4.2: Blank Solvent spectra of Acetone

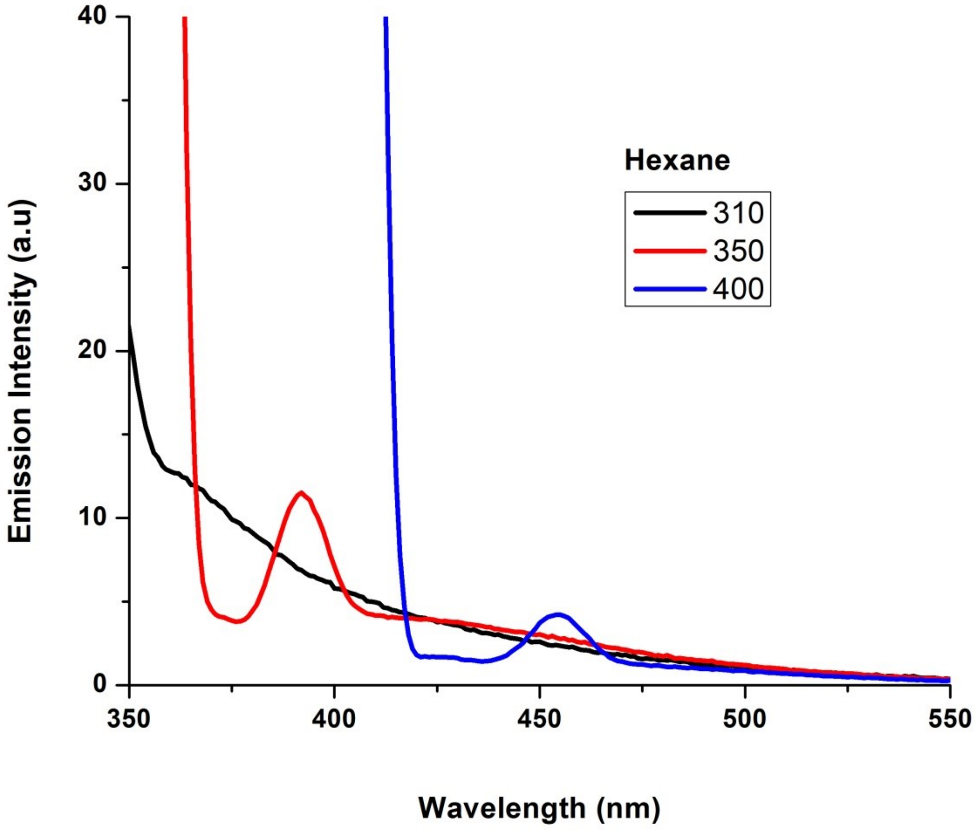

Figure S4.3: Blank Solvent spectra of Hexane
